# Supplementary material for: Interfacial alloying between lead halide perovskite crystals and hybrid glasses
Source: Nat Commun. 2023 Nov 22;14:7612. doi: 10.1038/s41467-023-43247-6 (PMC10665442; doi:10.1038/s41467-023-43247-6)
Supplement: Supplementary file 1 — Supplementary Information [file 41467_2023_43247_MOESM1_ESM.pdf]

Supplementary Information

Interfacial alloying between lead halide perovskite crystals and hybrid glasses

Li et al.

This PDF file includes:

Supplementary Table 1

Supplementary Figs.1 to 37

Supplementary Table 1. Results of the extended X-ray absorption fine structure (EXAFS) fitting for $(CsPbI_3)_{0.40}(a_gZIF\text{-}62)_{0.60}$ composites before and after 350°C sintering for Zn K and Pb L_3 edge.

Name	Coordination number (CN)	Bond length	Sintering	R-factor
Zn-N	5.07±0.26	1.99±0.004	Before	0.007
Zn-C	6.96±1.35	3.02±0.018	Before	0.007
Pb-O	3.27±0.35	2.46±0.007	Before	0.005
Pb-I	1.69±0.16	3.13±0.011	Before	0.005
Zn-N	5.04±0.31	1.98±0.005	After	0.007
Zn-C	4.59±1.10	2.99±0.020	After	0.007
Pb-I	6.80±1.23	3.12±0.042	After	0.010
Pb-Zn	4.08±0.47	1.80±0.013	After	0.010

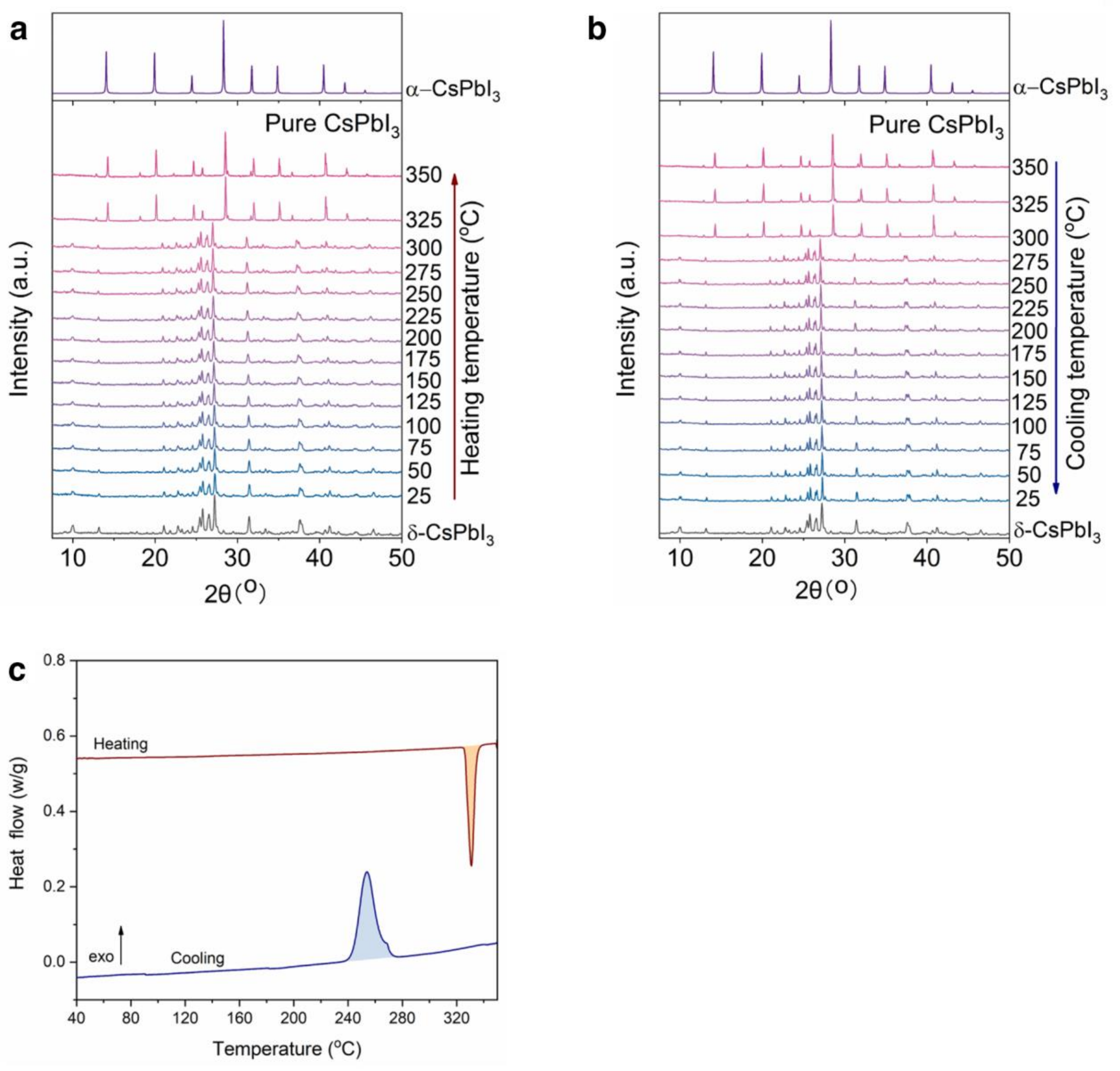

Supplementary Fig. 1. Phase transition of the pure $CsPbI_3$. a *In-situ* XRD pattern for pure $CsPbI_3$ during the heating process. **b** *In-situ* XRD pattern for pure $CsPbI_3$ during the cooling process. **c** DSC results for pure $CsPbI_3$ for heating and cooling ramps. Data was collected under constant flowing nitrogen protection (20 mL/min). The tempertaure ramping rate were 20 and 10 ℃/min during the heating and cooling process, respectively.

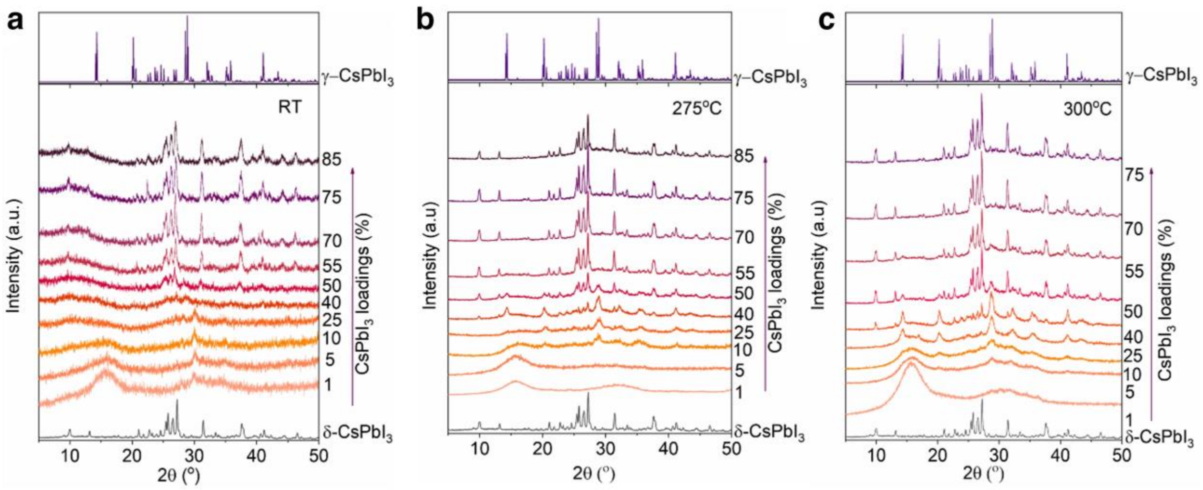

Supplementary Fig. 2. Crystal structures of the composites with different components and sintered under different temperatures. a *Ex-situ* XRD pattern of ($CsPbI_3$) (a_gZIF-62) (X/Y), and $(CsPbI_3)_X(a_gZIF\text{-}62)_Y$ composites prepared at **b** 275 ℃ and (c) 300 ℃ sintering.

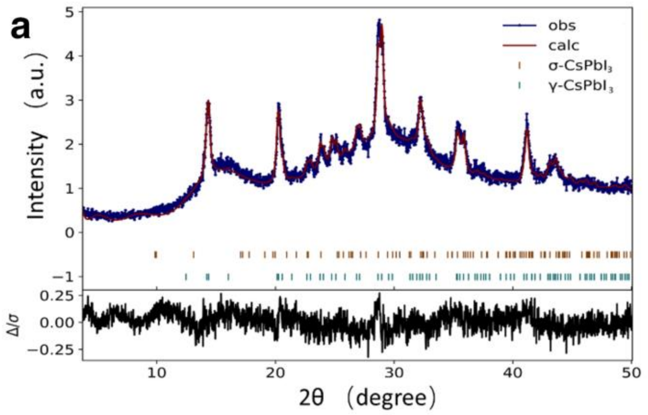

Lattice parameters	Lattice parameters reported in ref.	Lattice parameters	Lattice parameters reported in ref.
a=10.3822 Å	a=10.4500(5) Å	a=8.6232 Å	a=8.6198(6) Å
b=4.8068 Å	b=4.7965(2) Å	b=8.8085 Å	b=8.8518(6) Å
c=17.7523 Å	c=17.7602(8) Å	c=12.4534 Å	c=12.5012(7) Å
α=90°	α=90°	α=90°	α=90°
β=90°	β=90°	β=90°	β=90°
γ=90°	γ=90°	γ=90°	γ=90°
V=885.929 Å³	V=890.20(7) Å³	V=945.920 Å³	V=953.852(11) Å³
Space group: *Pnma*		Space group: *Pbnm*	
Crystal system: Orthorhombic (σ)		Crystal system: orthorhombic (γ)	
Weight faction: 0 (sig: 0.0078)		Weight faction: 100% (sig: 0.0078)	
R_{wp}=8.074%			

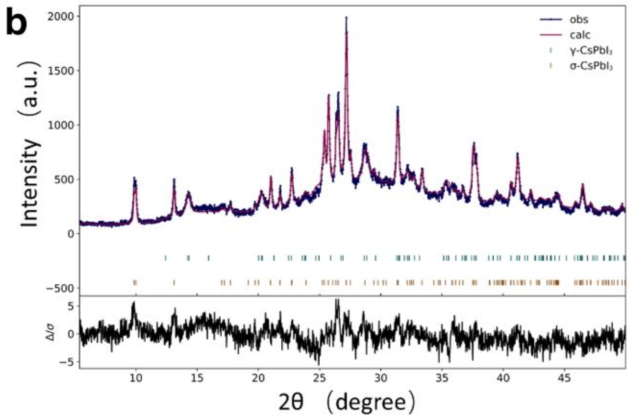

Lattice parameters	Lattice parameters reported in ref.	Lattice parameters	Lattice parameters reported in ref.
a=10.4382 Å	a=10.4500(5) Å	a=8.7326 Å	a=8.6198(6) Å
b=4.7884 Å	b=4.7965(2) Å	b=8.8679 Å	b=8.8518(6) Å
c=17.7395 Å	c=17.7602(8) Å	c=12.3619 Å	c=12.5012(7) Å
α=90°	α=90°	α=90°	α=90°
β=90°	β=90°	β=90°	β=90°
γ=90°	γ=90°	γ=90°	γ=90°
V=888.665 Å³	V=890.20(7) Å³	V=957.306 Å³	V=953.852(11) Å³
Space group: *Pnma*		Space group: *Pbnm*	
Crystal system: Orthorhombic (σ)		Crystal system: orthorhombic (γ)	
Weight faction: 71.31% (sig: 0)		Weight faction: 28.69% (sig: 0)	
R_{wp}=9.171%			

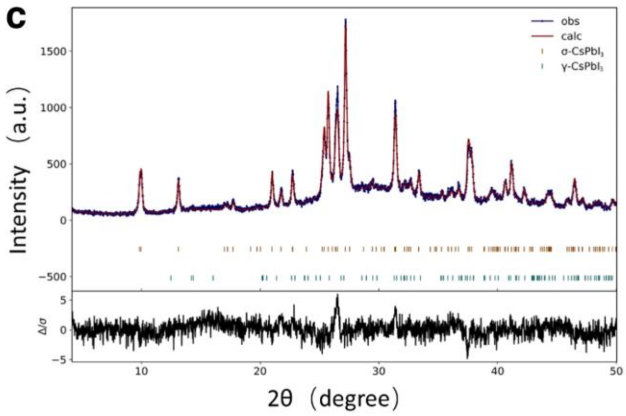

Lattice parameters	Lattice parameters reported in ref.	Lattice parameters	Lattice parameters reported in ref.
a=10.4493 Å	a=10.4500(5) Å	a=8.6447 Å	a=8.6198(6) Å
b=4.7966 Å	b=4.7965(2) Å	b=8.8178 Å	b=8.8518(6) Å
c=17.7603 Å	c=17.7602(8) Å	c=12.5002 Å	c=12.5012(7) Å
α=90°	α=90°	α=90°	α=90°
β=90°	β=90°	β=90°	β=90°
γ=90°	γ=90°	γ=90°	γ=90°
V=890.172 Å³	V=890.20(7) Å³	V=952.857 Å³	V=953.852(11) Å³
Space group: *Pnma*		Space group: *Pbnm*	
Crystal system: Orthorhombic (σ)		Crystal system: Orthorhombic (γ)	
Weight faction: 97.63% (sig: 0)		Weight faction: 2.37% (sig: 0)	
R_{wp}=8.612%			

Supplementary Fig. 3. Examples of identifying the proportions of various $CsPbI_3$ phases within composites using XRD refinement. a Rietveld refinement of powder XRD pattern of $(CsPbI_3)_{0.25}$ $(a_gZIF\text{-}62)_{0.75}$ composites sintering at 350 °C, **b** $(CsPbI_3)_{0.5}(a_gZIF\text{-}62)_{0.5}$ composites prepared at 300 °C and **c** $(CsPbI_3)_{0.70}(a_gZIF\text{-}62)_{0.30}$ composites sintering at 275°C.

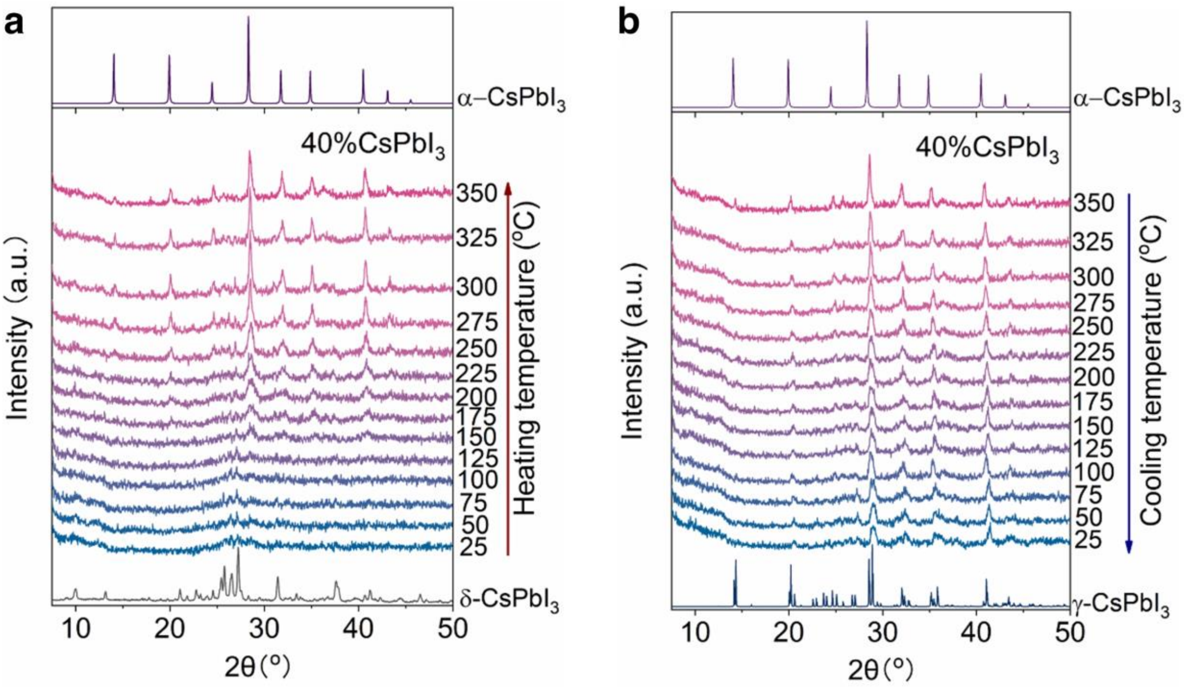

Supplementary Fig. 4. a *In-situ* XRD pattern of ($CsPbI_3$)(a_gZIF-62)(40/60) heated from 25 °C to 350 °C and **b** corresponding cooling down process. The temperature ramp is 20 °C/min under the protection of N_2. The reference XRD patterns of α-, γ- and δ-$CsPbI_3$ are retrieved from ref[1]. The intermediate β-phase was not resolved using our benchtop XRD instrument, due to resolution limits. During the cooling ramp, the perovskite gradually lowered its symmetry, resulting in the formation of the orthorhombic γ-phase upon returning to room temperature.

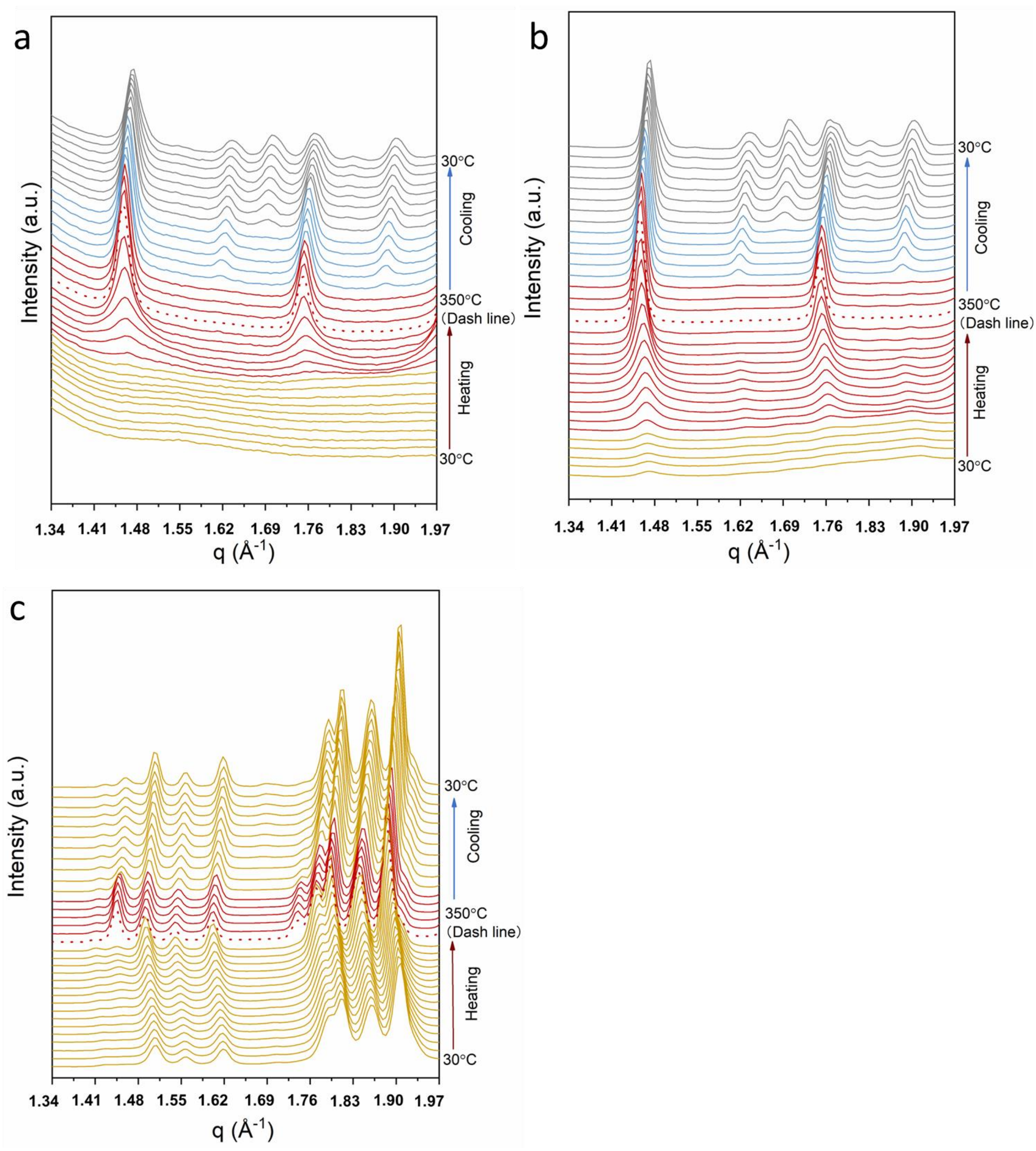

Supplementary Fig. 5. High resolution *in situ* synchrotron powder XRD for different samples during sintering and quenching. The dominating $CsPbI_3$ phases are color-coded as: δ (yellow), α (red), β (blue) and γ (grey). a ($CsPbI_3$)(a_gZIF-62)(5/95), **b** ($CsPbI_3$)(a_gZIF-62)(40/60) and **c** ($CsPbI_3$)(a_gZIF-62)(55/45).

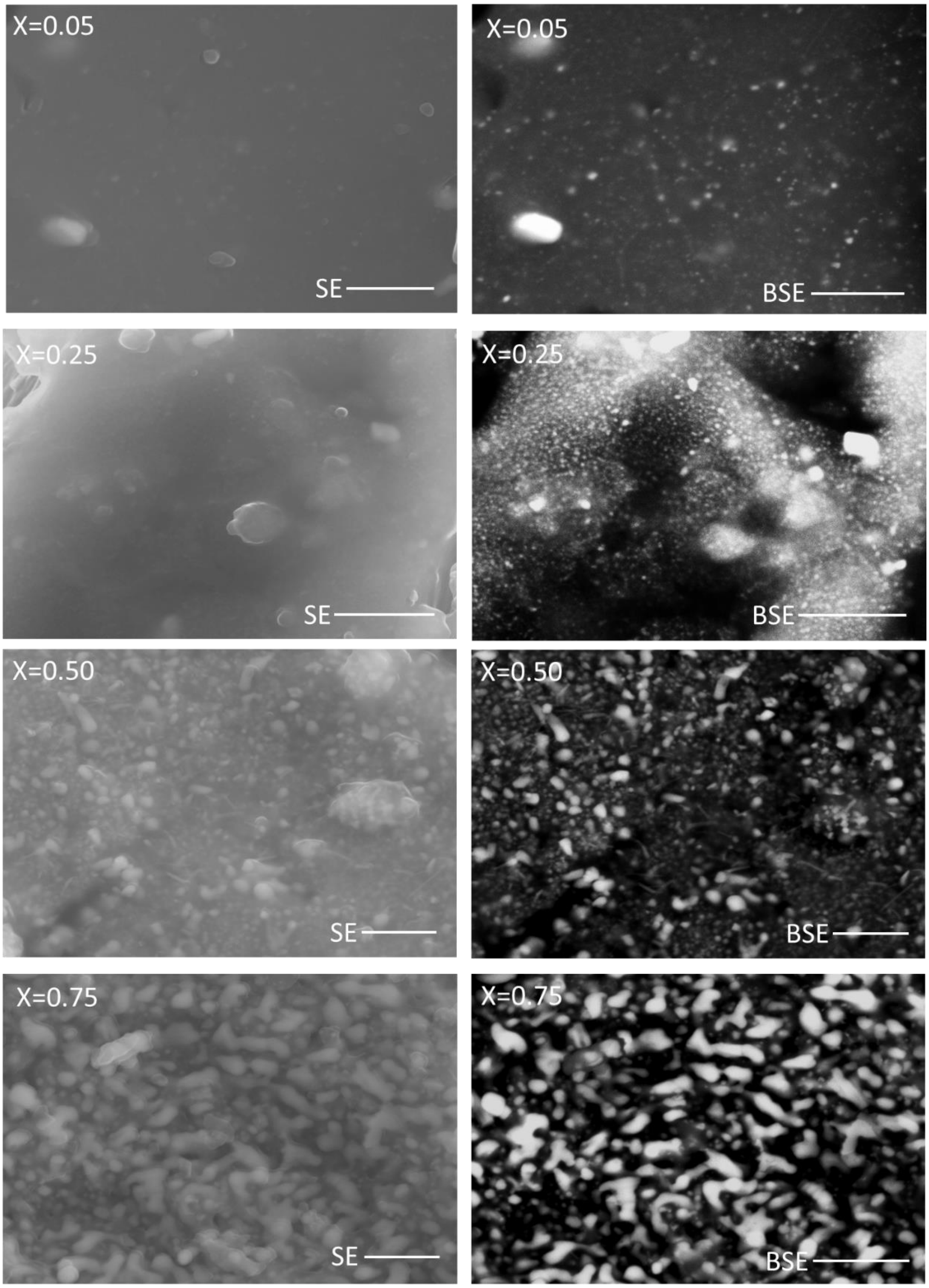

Supplementary Fig. 6. SEM secondary electron image (SE, left) and backscattering image (BSE, right) of $(CsPbI_3)_X(a_gZIF\text{-}62)_Y$ composites sintered at 350 °C. The scale bar is 1μm.

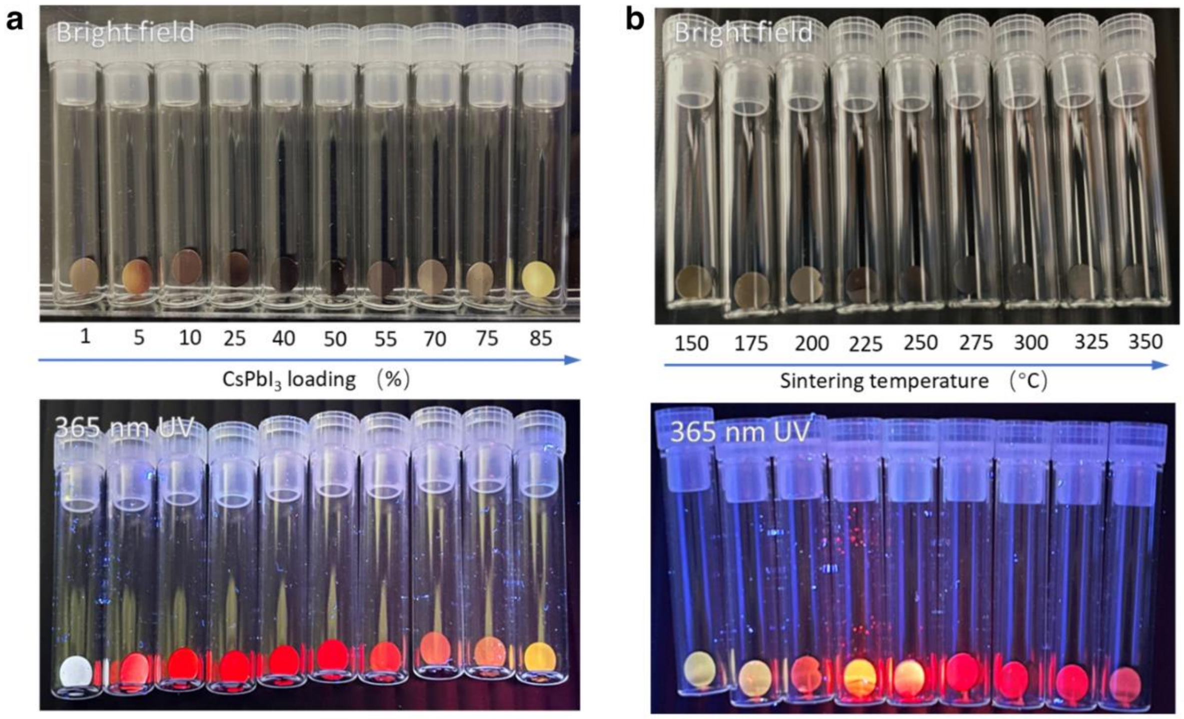

Supplementary Fig. 7. PL emission of the composites. a Optical images of the $(CsPbI_3)_X(a_gZIF\text{-}62)_Y$ composites sintering at 275 ℃ with different $CsPbI_3$ components and **b** $(CsPbI_3)_{0.25}(a_gZIF\text{-}62)_{0.75}$ composites with various sintering temperatures.

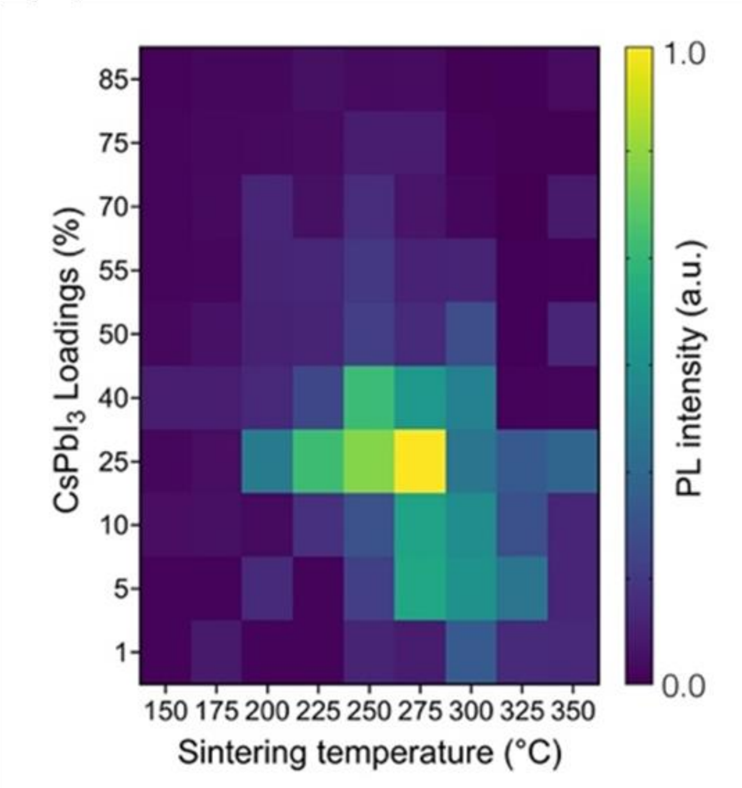

Supplementary Fig. 8. Relative PL intensity of different $(CsPbI_3)_X(a_gZIF\text{-}62)_Y$ composites.

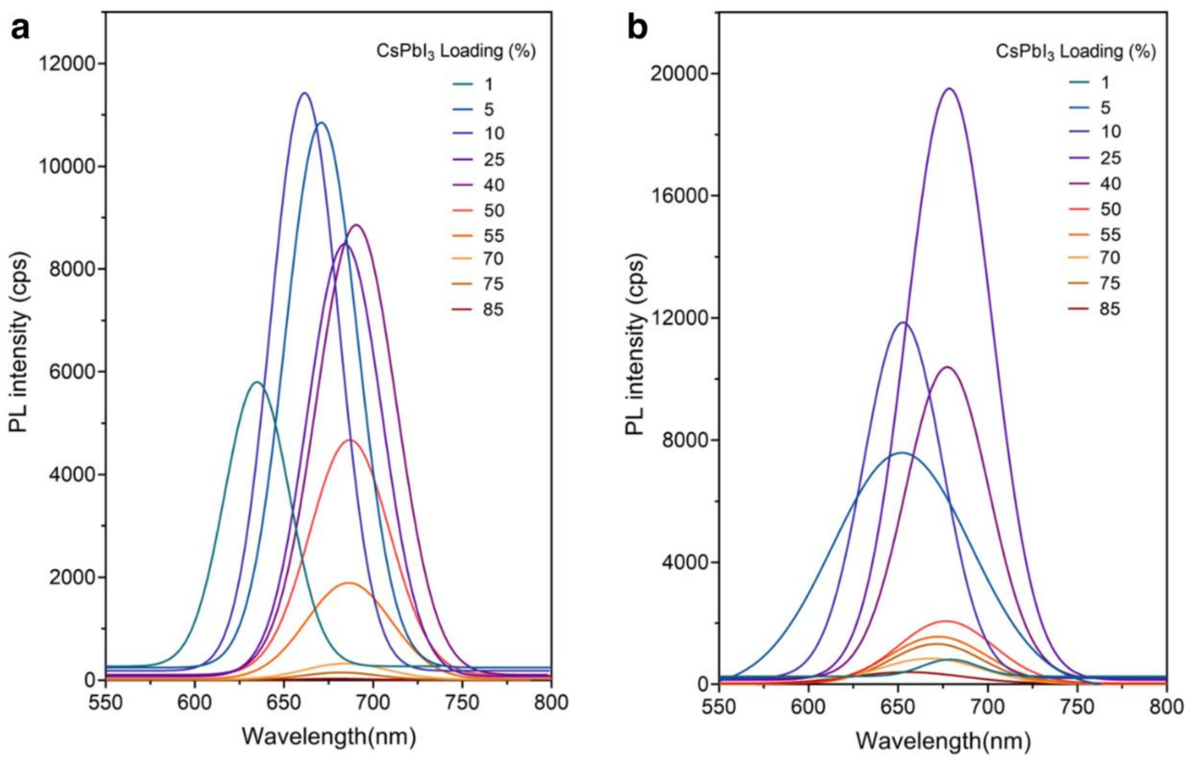

Supplementary Fig. 9. PL profiles of different $CsPbI_3$ loading samples sintered at a 300°C and b 275°C.

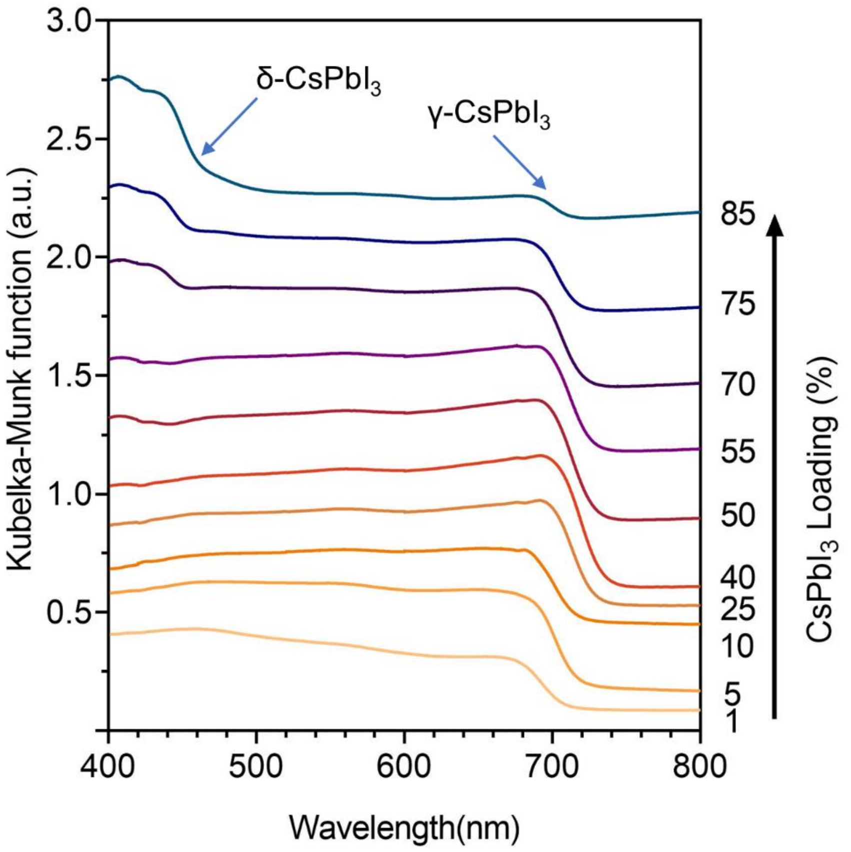

Supplementary Fig. 10. UV-Vis absorption spectra for different $CsPbI_3$ percentages composites sintered at 300 ℃.

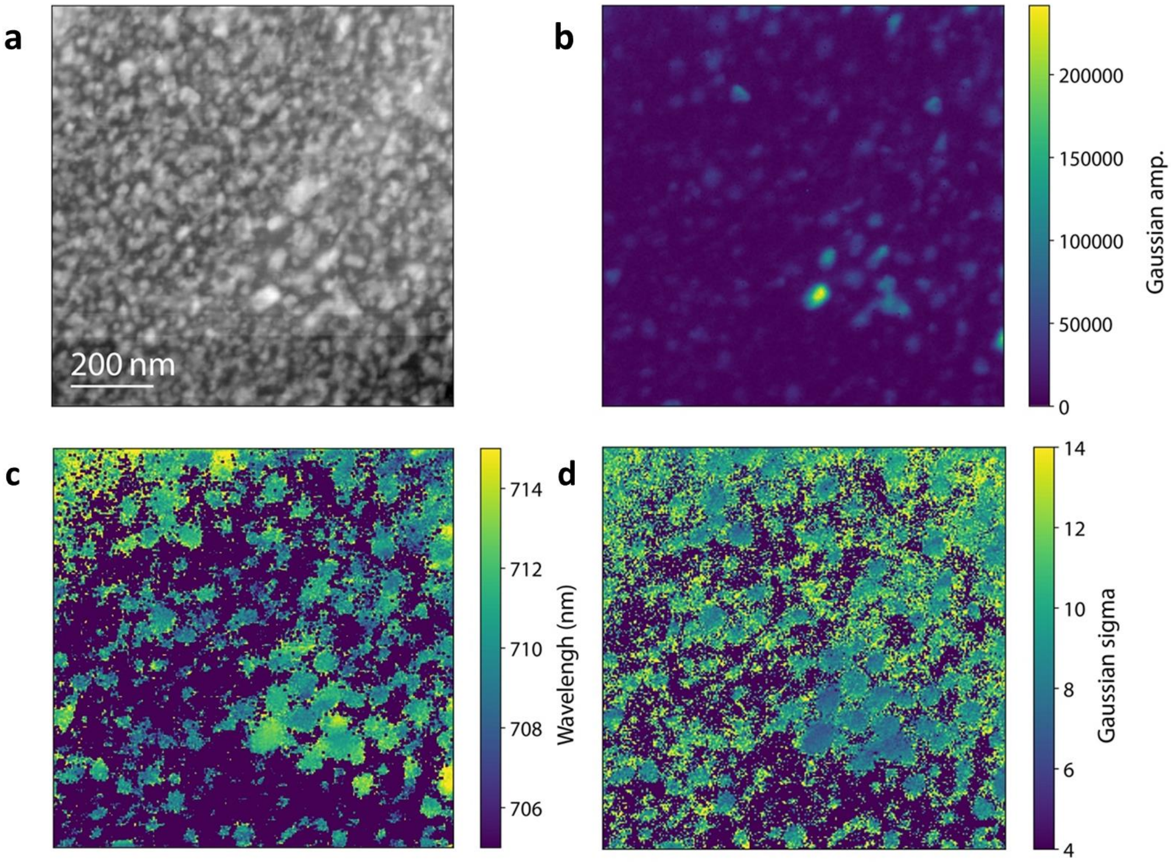

Supplementary Fig. 11. Cathodoluminescence (CL)-STEM characterisation of the $(CsPbI_3)_{0.40}(a_gZIF\text{-}62)_{0.60}$ composites sintered at 350 °C and thinned with FIB-SEM. a ADF-STEM image. **b** Mapping of the Gaussian fitted CL peak intensity. **c** Mapping of the Gaussian fitted CL peak position values and **d** Gaussian fitted σ (nm) values.

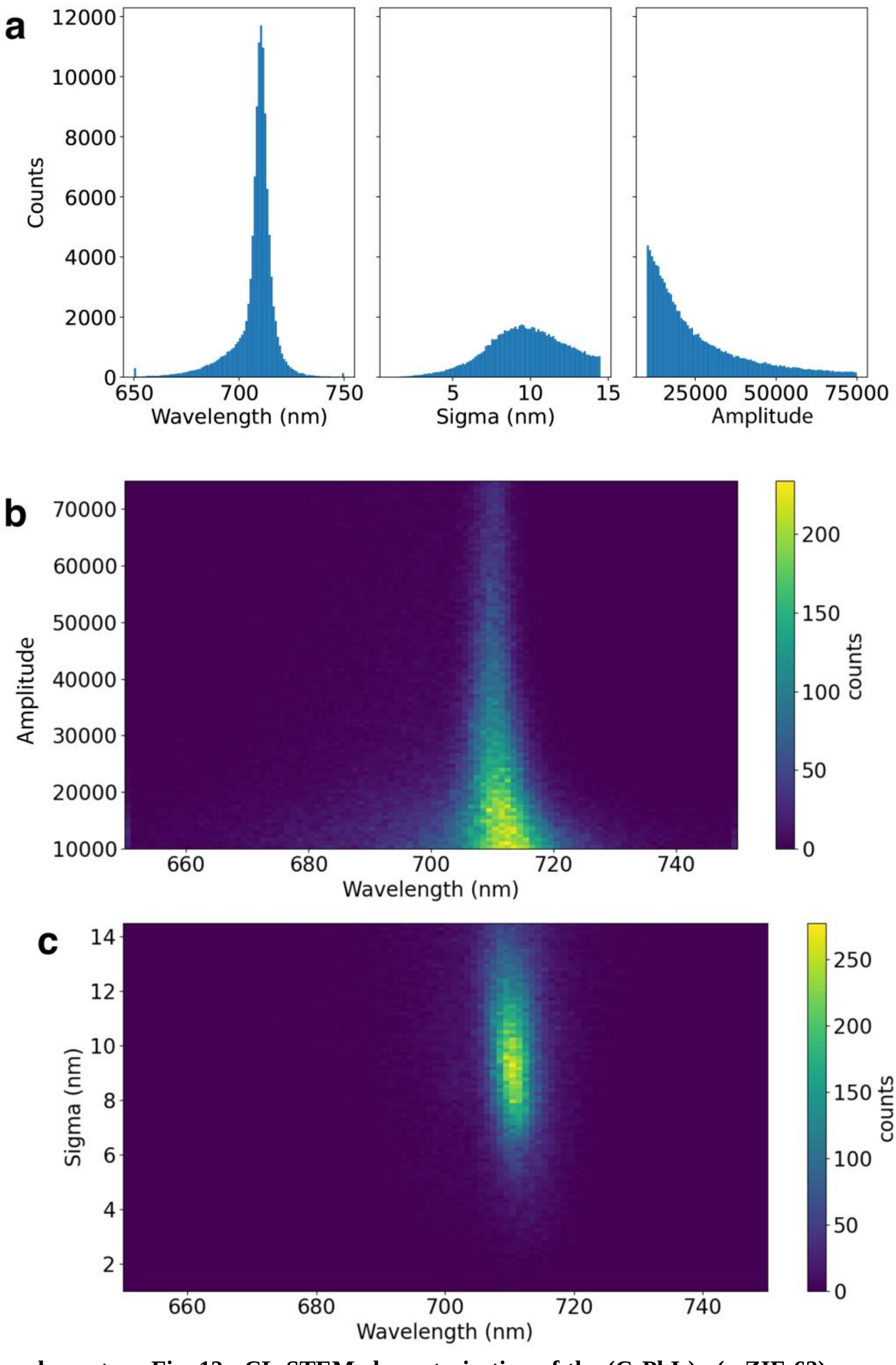

Supplementary Fig. 12. CL-STEM characterisation of the $(CsPbI_3)_{0.1}(a_gZIF\text{-}62)_{0.9}$ composites sintered at 350 °C. CL data cubes have been acquired from a total project area of 6.9 µm^2. A one Gaussian model was fitted to each spectrum. **a** Histograms of the fitted coefficients across the whole measured area: Gaussian peak center, sigma and amplitude. **b, c** 2D histogram of the amplitude versus wavelength and sigma versus wavelength shows that correlations exist. Lower wavelength peaks are associated to higher intensities.

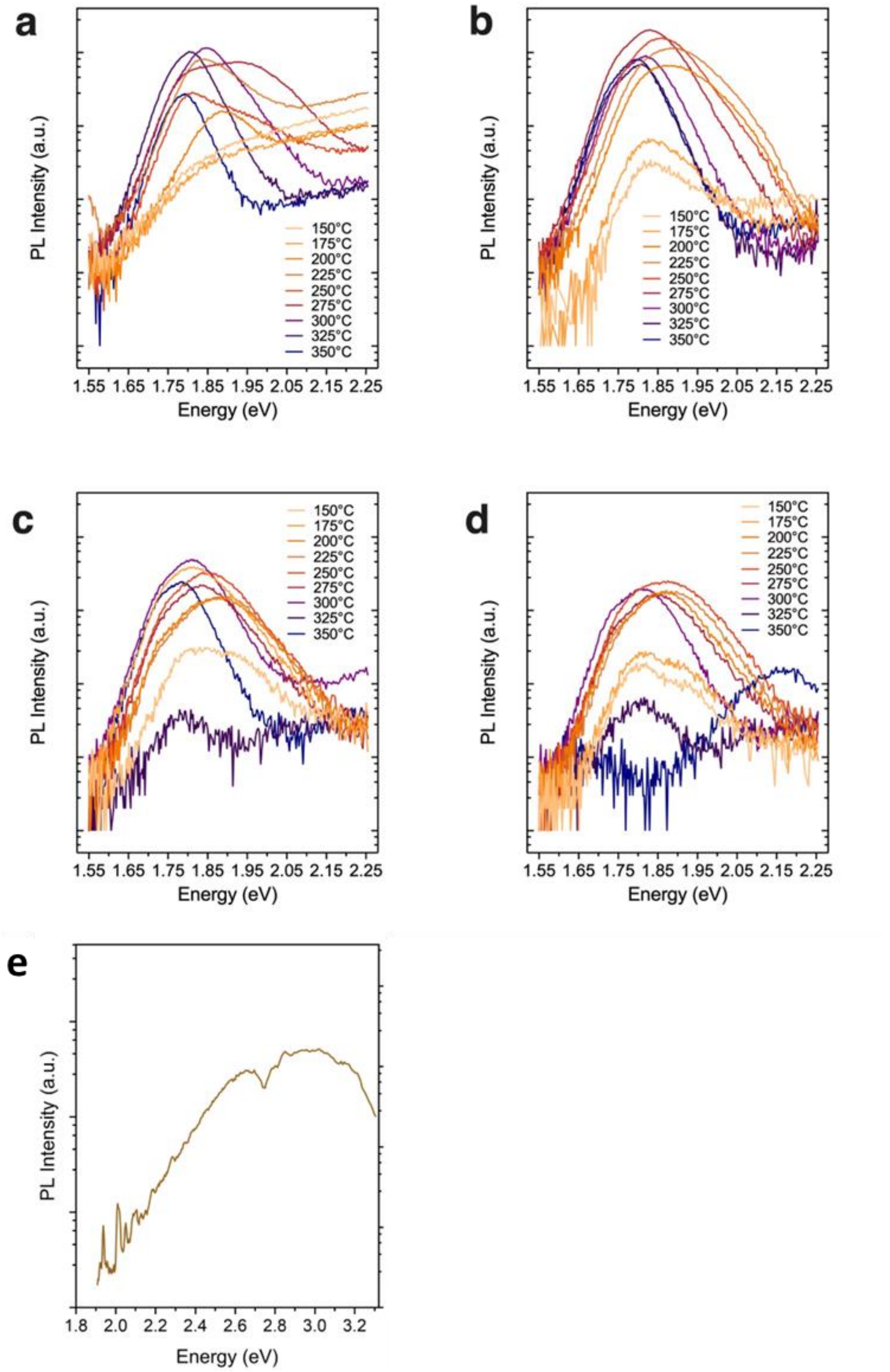

Supplementary Fig. 13. PL spectra shown in log scale for a $(CsPbI_3)_{0.05}(a_gZIF\text{-}62)_{0.95}$ composite, b $(CsPbI_3)_{0.25}(a_gZIF\text{-}62)_{0.75}$ composite, c $(CsPbI_3)_{0.50}(a_gZIF\text{-}62)_{0.50}$ composite, d $(CsPbI_3)_{0.55}(a_gZIF\text{-}62)_{0.45}$ composite and (e) pure $a_gZIF\text{-}62$.

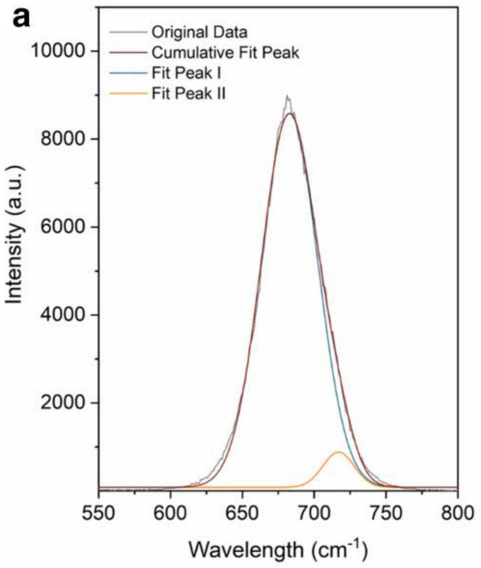

b

Model	Gauss	
Equation	$y=y_0+(A/(w*sqrt(pi/2)))*exp(-2*((x-x_c)/w)^2)$	
Plot	Peak I	Peak II
y_0	81.13 ± 9.77	81.13 ± 9.77
x_c	682.82 ± 0.20	716.94 ± 0.97
w	39.27 ± 0.33	21.78 ± 1.75
A	418305.79 ± 3918.98	21896.09 ± 3476.46
Reduced Chi-Sqr	13025.42	
R-Square (COD)	0.998	
Adj. R-Square	0.998	

Supplementary Fig. 14. Representative PL fitting with two-Gaussian function. a PL curve of $(CsPbI_3)_{0.25}(a_gZIF\text{-}62)_{0.75}$ composite sintering at 300 ℃ fitted by two peaks Gaussian fits, the peaks were noted as Peak I and Peak II. **b** The summary of fitting results and peak parameters. Peak I represents the high energy peak and the Peak II represents the low energy peak.

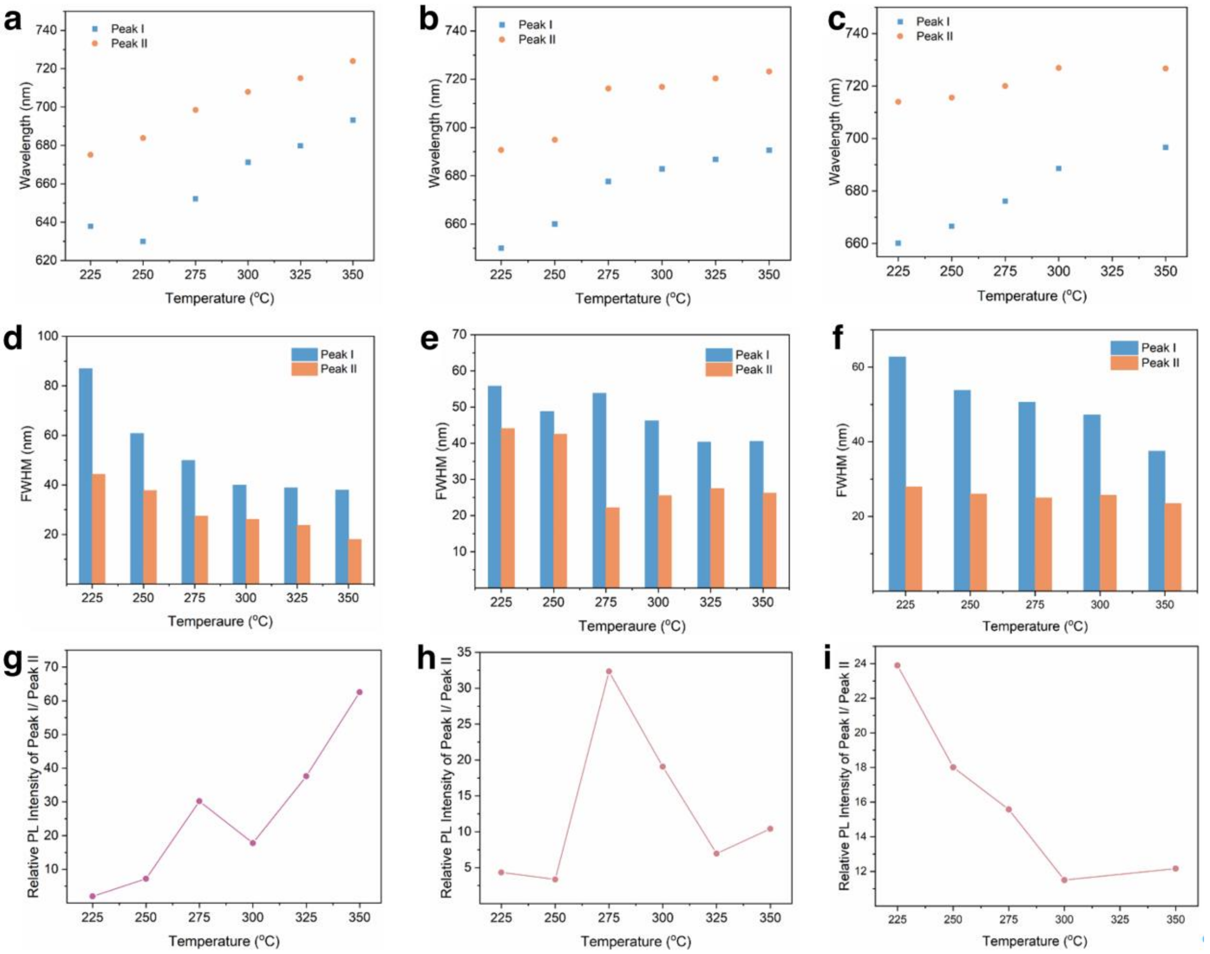

Supplementary Fig. 15. Summary of the two-Gaussian fitting of PL, where Peak I represents the high energy peak and the Peak II represents the low energy peak. The extracted peak positions for **a** $(CsPbI_3)_{0.1}(a_gZIF\text{-}62)_{0.9}$, **b** $(CsPbI_3)_{0.25}(a_gZIF\text{-}62)_{0.75}$ and **c** $(CsPbI_3)_{0.40}(a_gZIF\text{-}62)_{0.60}$ composites. The summary of peak bandwidths from **d** $(CsPbI_3)_{0.1}(a_gZIF\text{-}62)_{0.9}$, **e** $(CsPbI_3)_{0.25}(a_gZIF\text{-}62)_{0.75}$ and **f** $(CsPbI_3)_{0.40}(a_gZIF\text{-}62)_{0.60}$ composites. The relative intensity of Peak I and Peak II from **g** $(CsPbI_3)_{0.1}(a_gZIF\text{-}62)_{0.9}$, **h** $(CsPbI_3)_{0.25}(a_gZIF\text{-}62)_{0.75}$ and **i** $(CsPbI_3)_{0.40}(a_gZIF\text{-}62)_{0.60}$ composites.

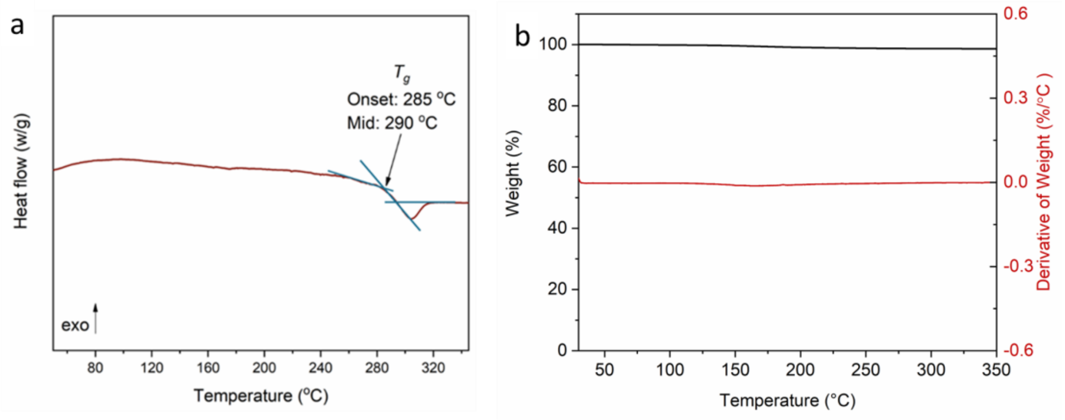

Supplementary Fig. 16. DSC and TGA results upon sintering to 350 ℃. Data was collected under constant flowing nitrogen (20 mL/min). a DSC results for melt-quenched a_gZIF-62 and **b** TGA results for $(CsPbI_3)$(a_gZIF-62)(40/60)**.** The temperature ramp rate was 20 °C /min.

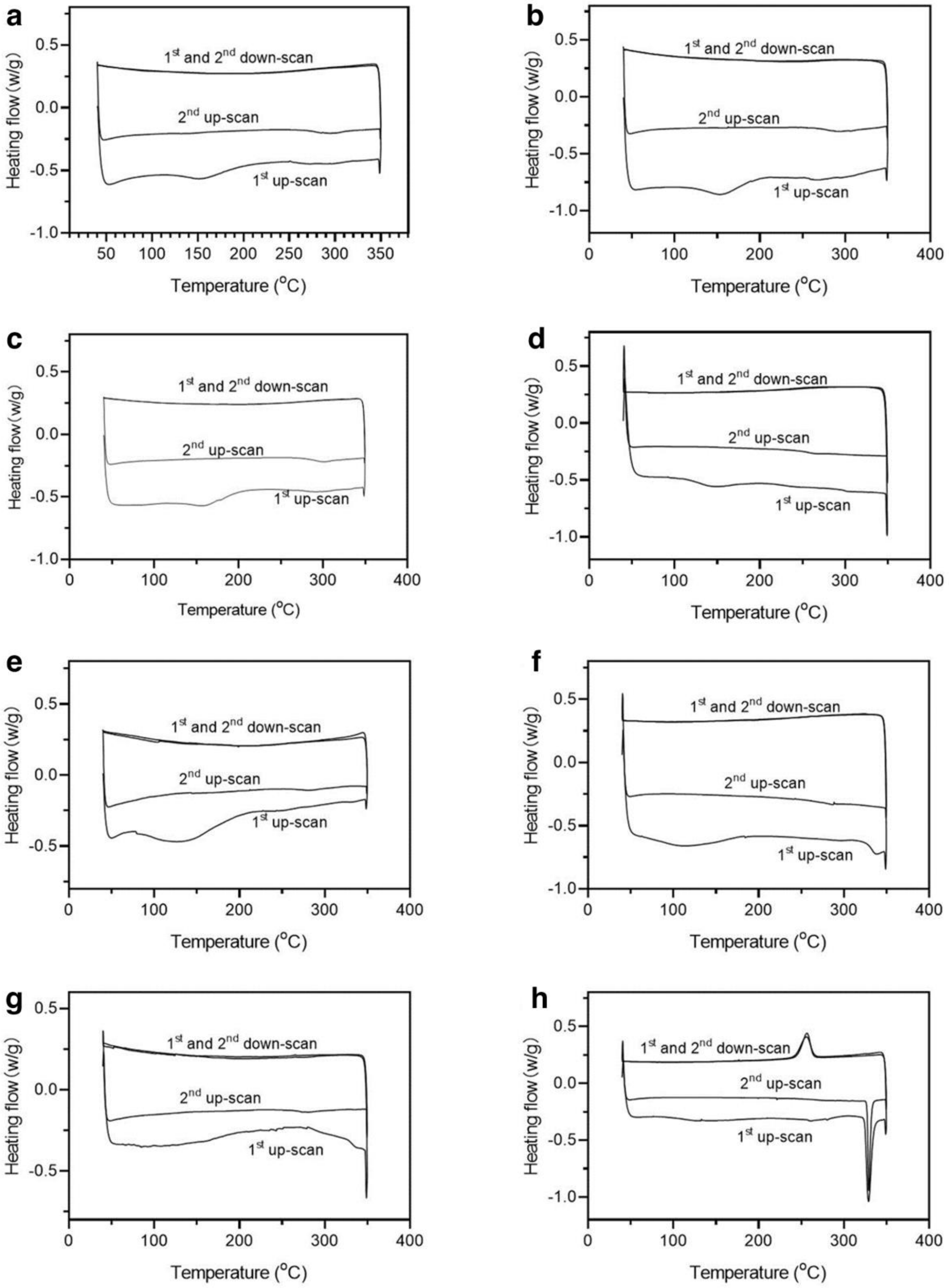

Supplementary Fig. 17. DSC data of $(CsPbI_3)_x(a_gZIF\text{-}62)_Y$ composites with different $CsPbI_3$ loadings. a $CsPbI_3)_{0.01}(a_gZIF\text{-}62)_{0.99}$, **b** $(CsPbI_3)_{0.05}(a_gZIF\text{-}62)_{0.95}$, **c** $(CsPbI_3)_{0.10}(a_gZIF\text{-}62)_{0.90}$, **d** $(CsPbI_3)_{0.25}(a_gZIF\text{-}62)_{0.75}$, **e** $CsPbI_3)_{0.40}(a_gZIF\text{-}62)_{0.60}$, **f** $CsPbI_3)_{0.50}(a_gZIF\text{-}62)_{0.50}$, **g** $CsPbI_3)_{0.55}(a_gZIF\text{-}62)_{0.45}$ and **h** $CsPbI_3)_{0.75}(a_gZIF\text{-}62)_{0.25}$ composites. The temperature ramping rate for the first up-scan was 20 ℃/min, and the ramping rate was 10 ℃/min during the DSC cooling and second up-scan.

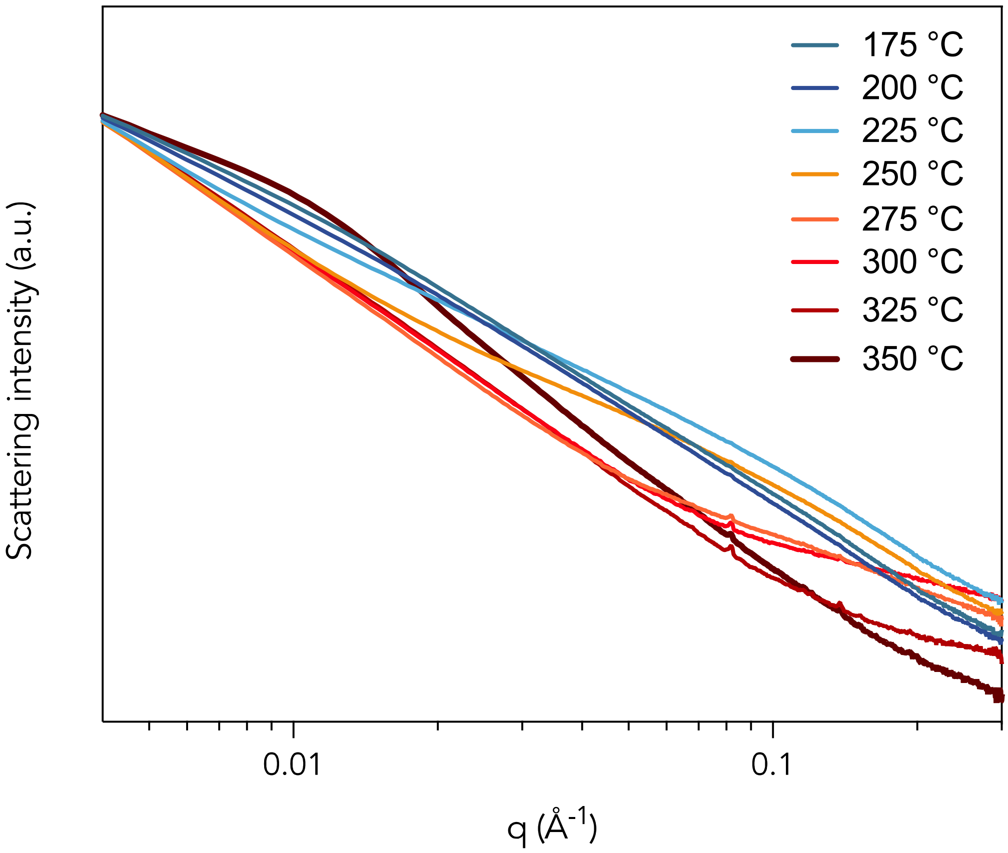

Supplementary Fig. 18. ***Ex-situ*** **synchrotron small angel scattering (SAXS) of $(CsPbI_3)_{0.1}(agZIF\text{-}62)_{0.9}$ fabricated with different sintering temperatures.** The spike at *ca.* 0.08 $Å^{-1}$ is attributed to the detector configuration.

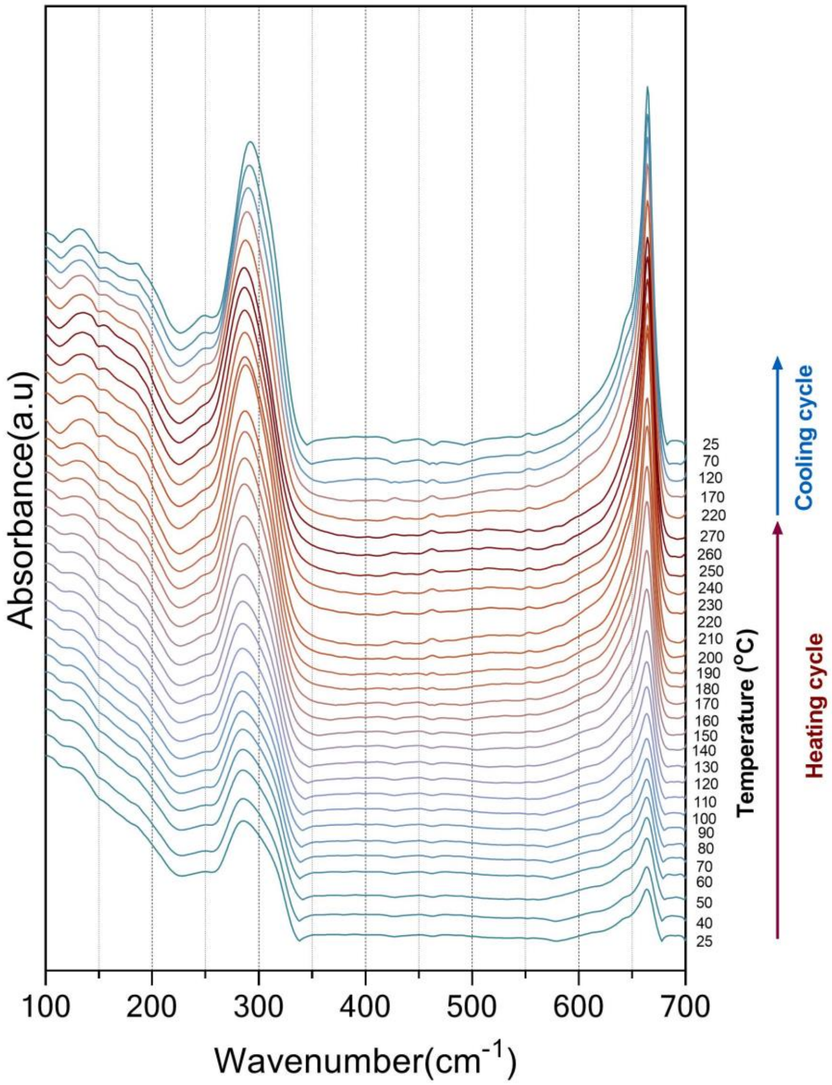

Supplementary Fig. 19. Temperature-resolved *in-situ* THz FarIR spectra for ($CsPbI_3$)(a_gZIF-62)(10/90) composites during heating and cooling ramps. Ramp rate was 10°C/min.

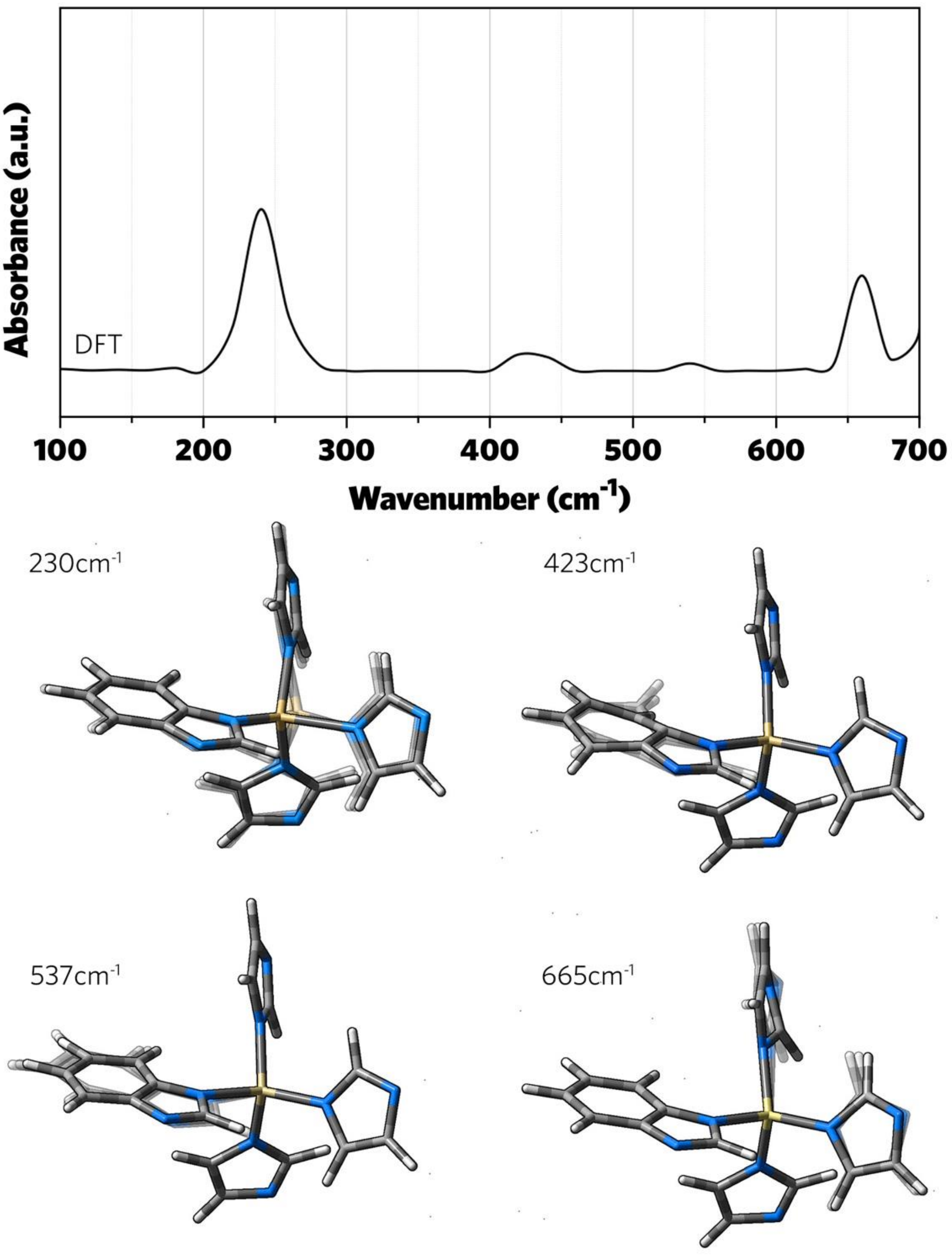

Supplementary Fig. 20. DFT calculations of the ZIF-62 THz absorbance spectrum. The scheme represents the vibration and deformation movement of the corresponding features. More detailed DFT calculation procedures can be found in our previous publication[2].

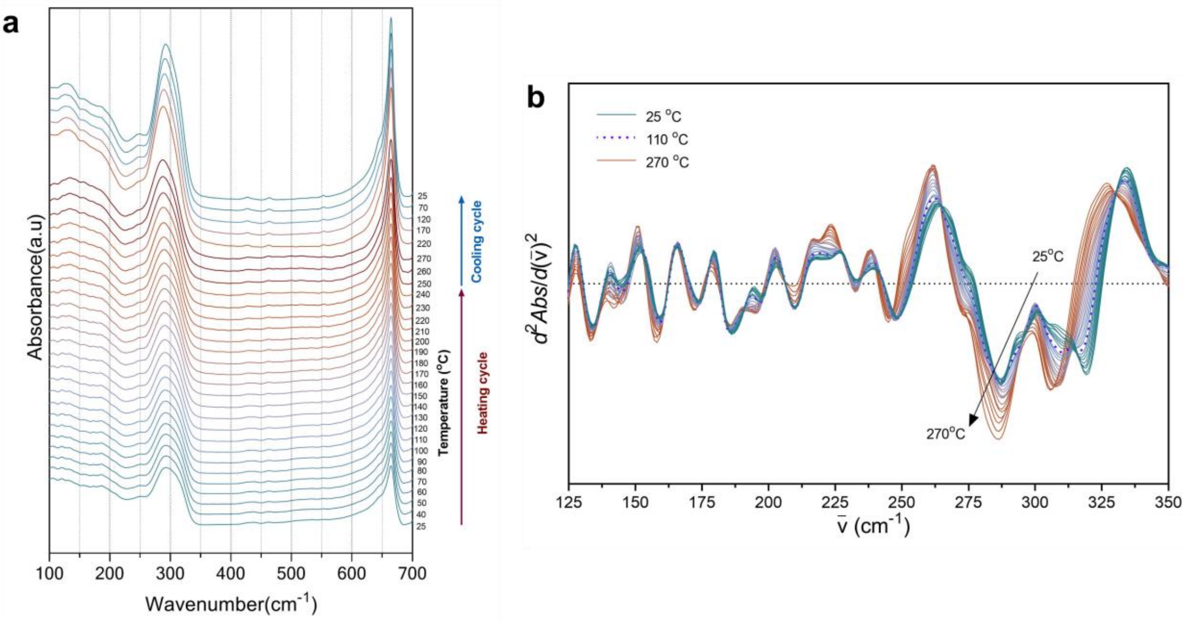

Supplementary Fig. 21. a Temperature-resolved *in-situ* THz FarIR spectra for $(CsPbI_3)(a_gZIF\text{-}62)(1/99)$ composites and b corresponding second derivative spectra. The sample were heated to 270 ℃ and cooling back to room temperature under Ar protections.

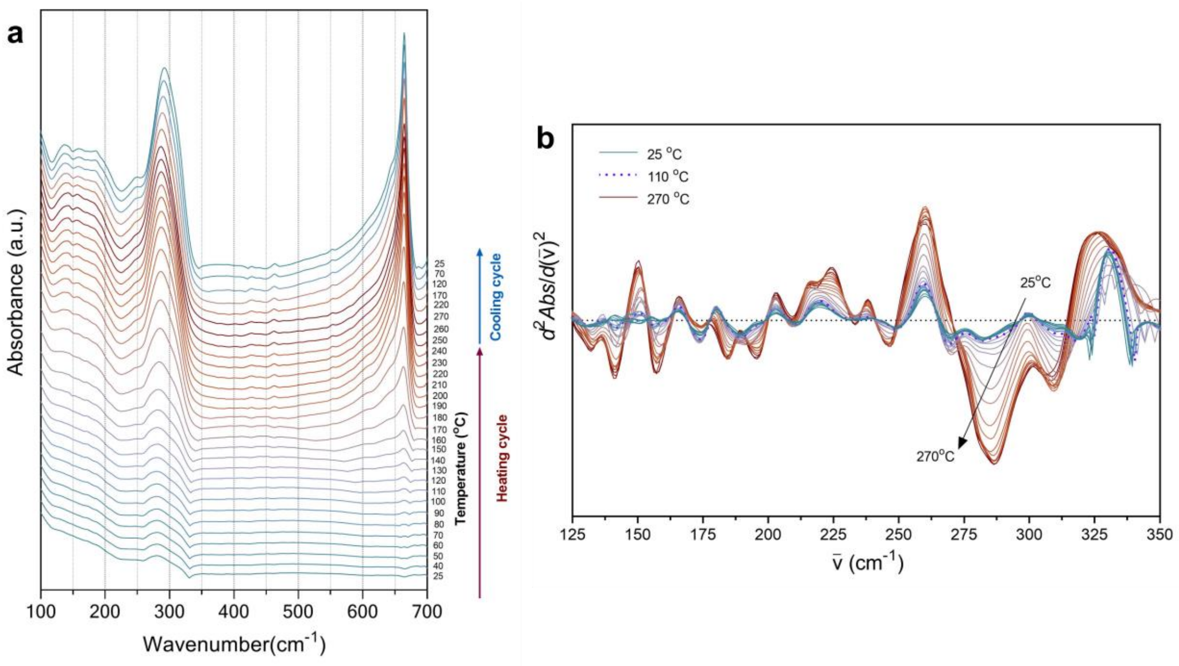

Supplementary Fig. 22. a Temperature-resolved *in-situ* THz FarIR spectra for ($CsPbI_3$)(a_gZIF-62)(25/75) composites and b corresponding second derivative spectra. The sample were heated to 270 ℃ and cooling back to room temperature under Ar protections.

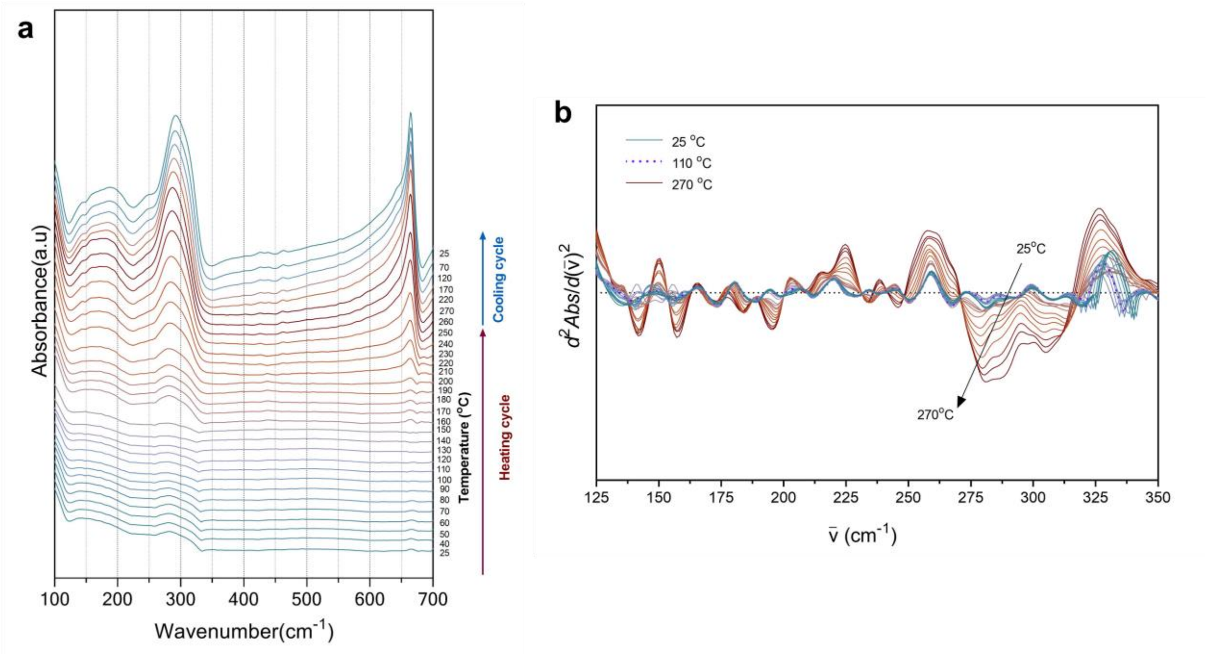

Supplementary Fig. 23. a Temperature-resolved *in-situ* THz FarIR spectra for ($CsPbI_3$)(a_gZIF-62)(55/45) composites and b corresponding second derivative spectra. The sample were heated to 270 ℃ and cooling back to room temperature under Ar protections.

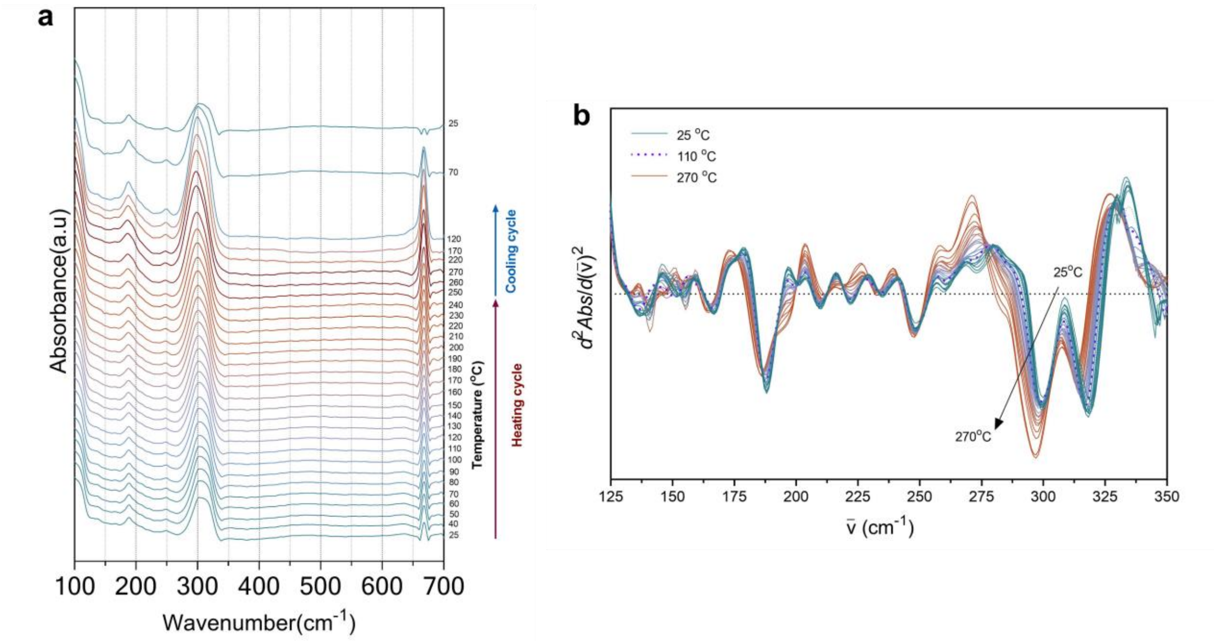

Supplementary Fig. 24. a Temperature-resolved *in-situ* THz FarIR spectra for ($CsPbI_3$)(a_gZIF-62)(85/15) composites and b corresponding second derivative spectra. The sample were heated to 270 ℃ and cooling back to room temperature under Ar protections.

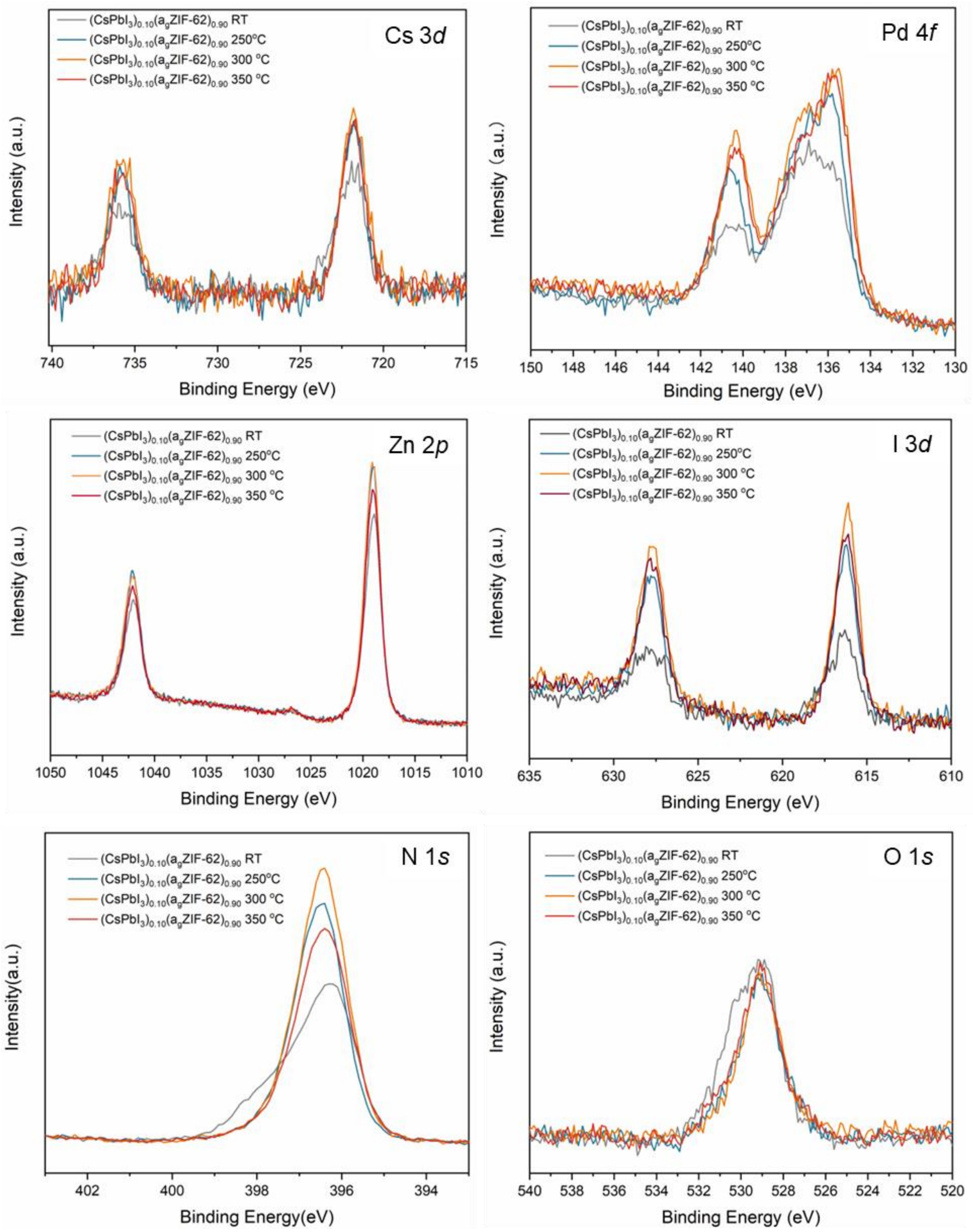

Supplementary Fig. 25. X-ray photoelectron spectroscopy (XPS) of $(CsPbI_3)_{0.1}(a_gZIF\text{-}62)_{0.9}$ composites at different sintering temperatures.

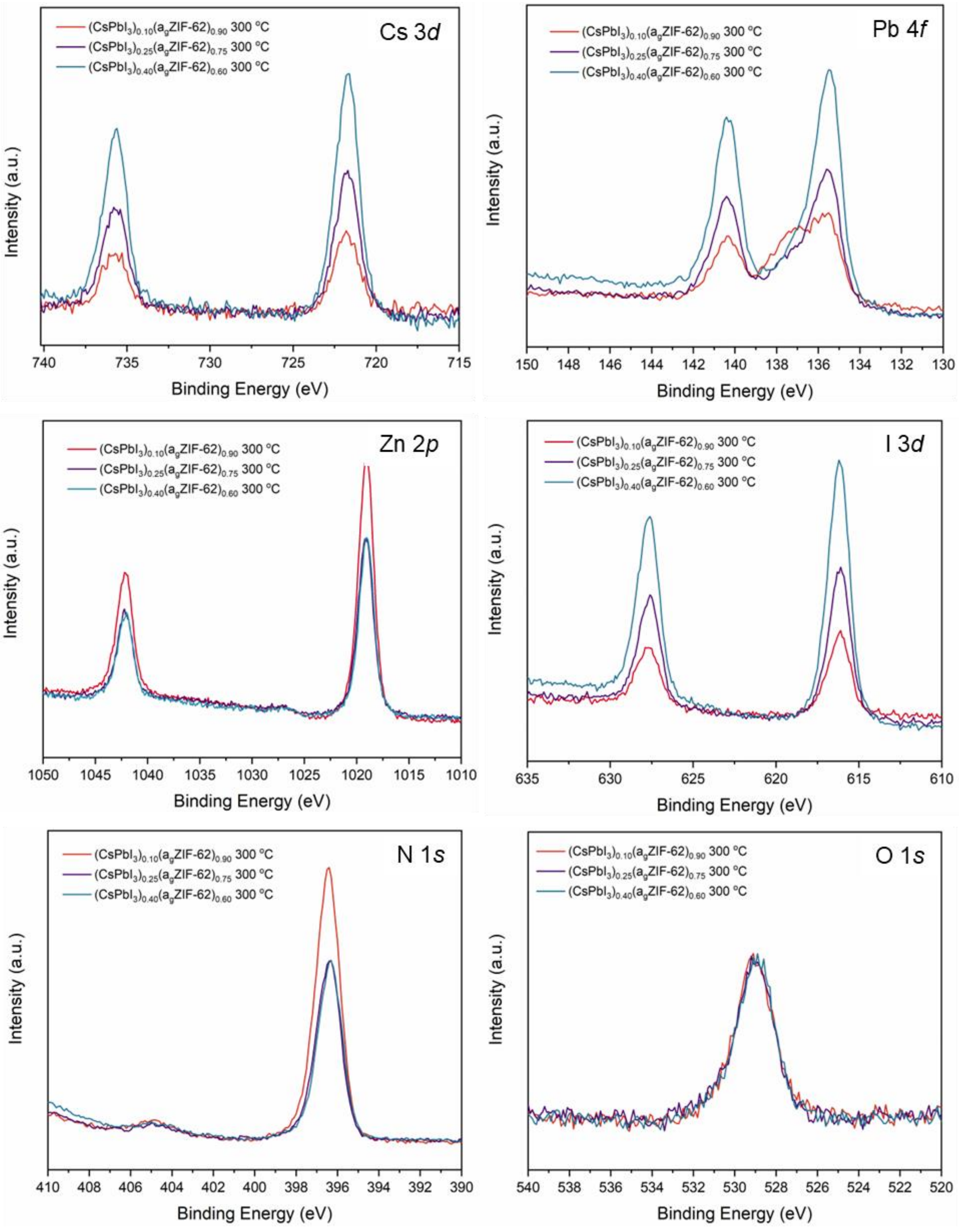

Supplementary Fig. 26. X-ray photoelectron spectroscopy (XPS) of $(CsPbI_3)_X(a_gZIF\text{-}62)_Y$ composites sintering at 300 °C with different components.

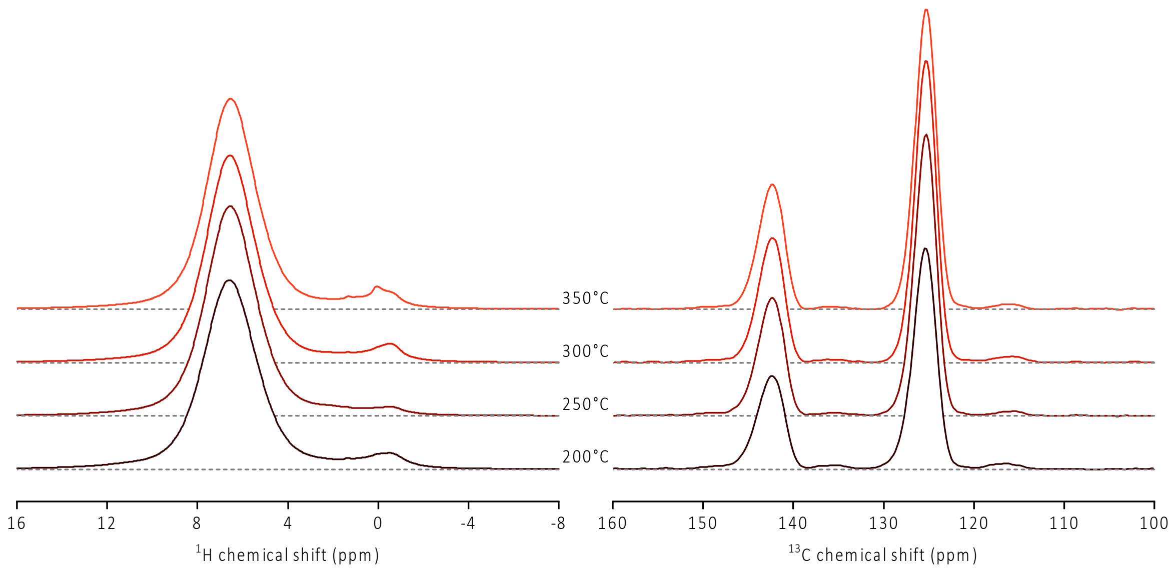

Supplementary Fig. 27. ^{1}H and ^{13}C MAS NMR spectra of $(CsPbI_3)_{0.25}(a_gZIF\text{-}62)_{0.75}$ composites sintered at 200, 250, 300, and 350 °C.

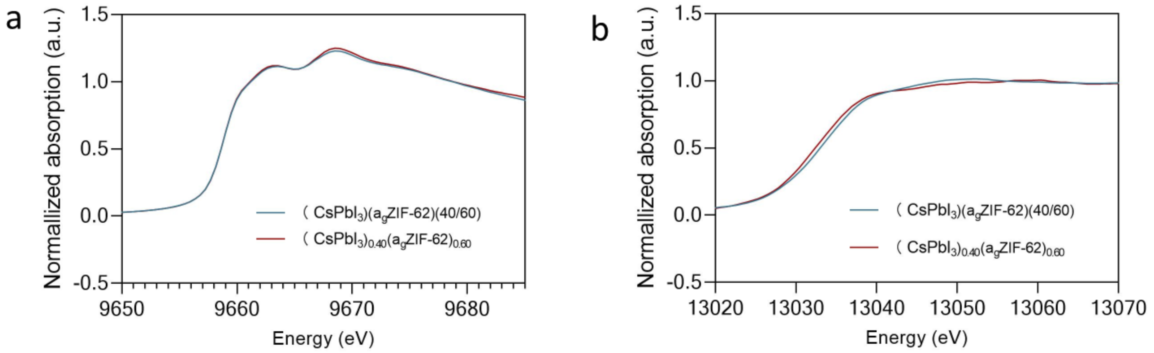

Supplementary Fig. 28. X-ray absorption of 40 wt% $CsPbI_3$ / a_gZIF-62 composites prior and after 350 °C sintering. **a** Zn K edge X-ray absorption spectrum near edge structure (XANES) and **b** Pb L_3-edge XANES.

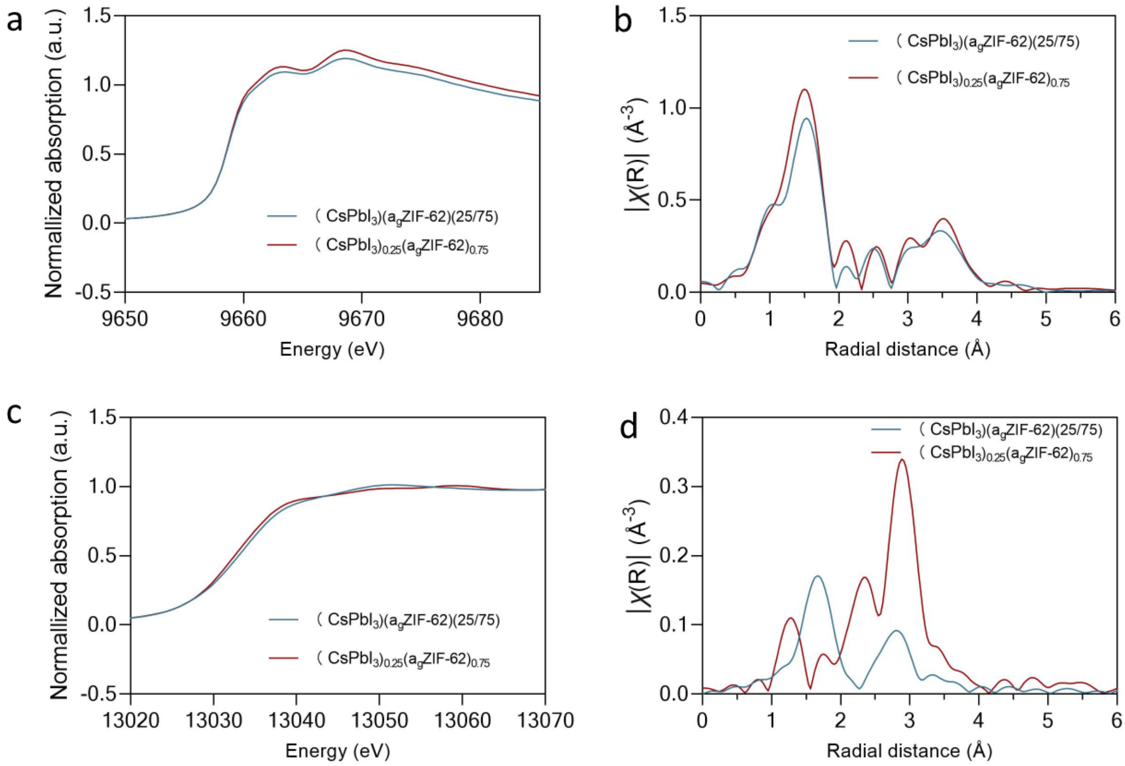

Supplementary Fig. 29. X-ray absorption of 25 wt% $CsPbI_3$ / a_gZIF-62 composites prior and after 350 °C sintering. a Zn edge X-ray absorption spectrum near edge structure (XANES) and **b** correspondent phase-uncorrected moduli of the Fourier transform results. **c** Pb edge X-ray absorption spectrum near edge structure (XANES) and **d** correspondent phase-uncorrected moduli of the Fourier transform results.

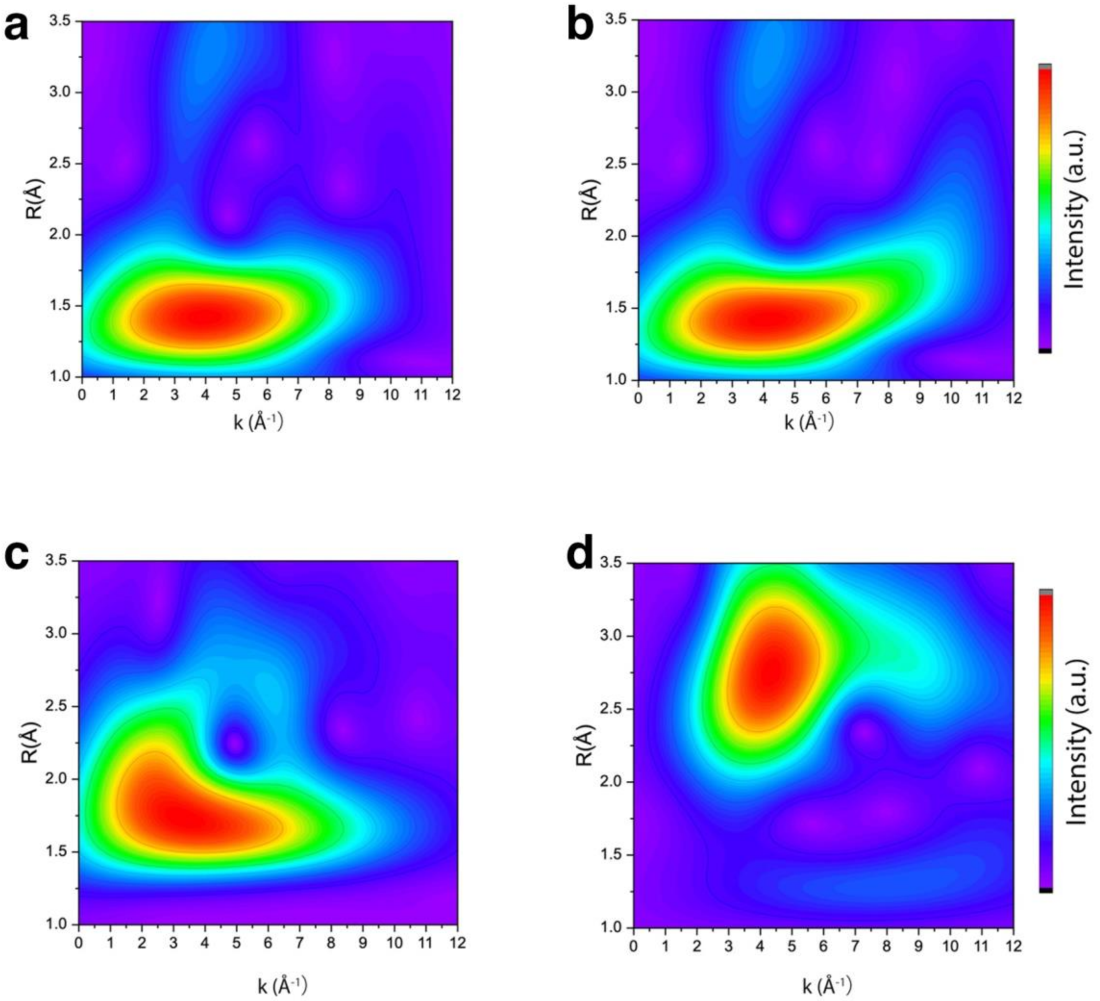

Supplementary Fig. 30. Full-range wavelet transform representation of the EXAFS signal for the $(CsPbI_3)_{0.25}(a_gZIF\text{-}62)_{0.75}$ composites. **a** Zn and **c** Pb represent the sample prior to sintering, and **b** Zn and **d** Pb represent the sample after 350 °C sintering.

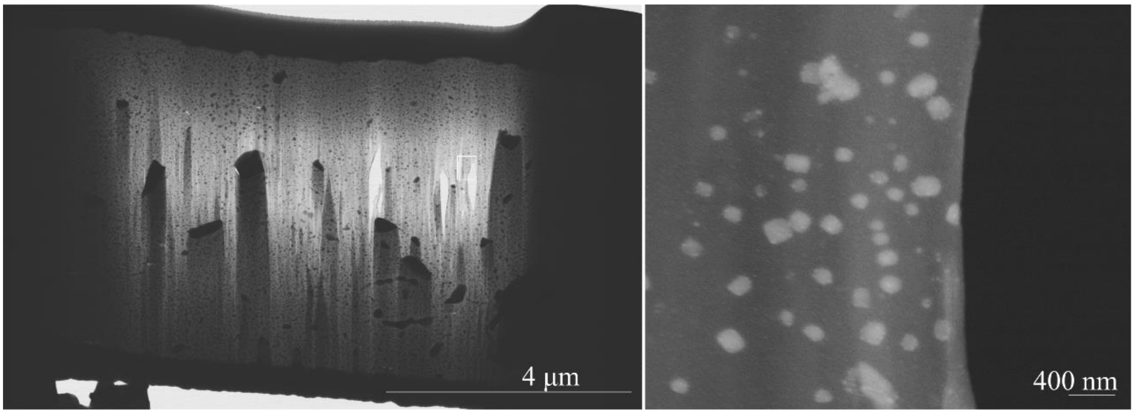

Supplementary Fig. 31. FIB-SEM images for $(CsPbI_3)_{0.25}(a_gZIF\text{-}62)_{0.75}$ composites sintering 350 °C.

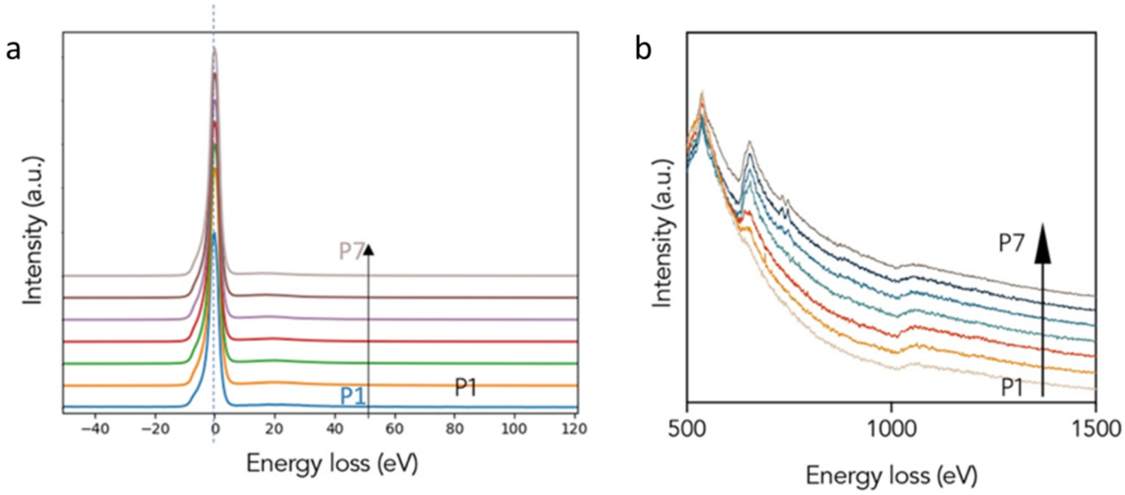

Supplementary Fig. 32. Data processing to for the TEM-EELS results of $(CsPbI_3)_{0.1}(agZIF\text{-}62)_{0.9}$ composites sintered at 350 °C. a Zero loss peak: normalised to total low loss scattering (plot with Y-offsets) and **b** Core loss: Normalised to total core loss scattering (plot with Y-offsets)

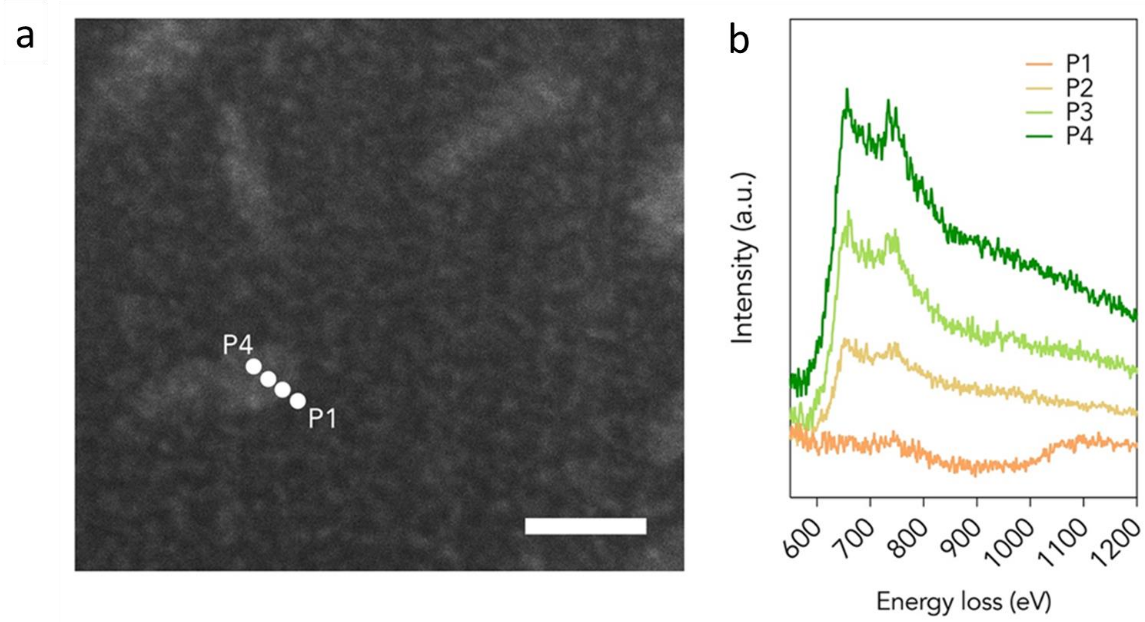

Supplementary Fig. 33. a Bright field STEM image of $(CsPbI_3)_{0.1}(a_gZIF\text{-}62)_{0.9}$ composites sintered at 275 °C. Scale bar, 50 nm. b EELS spectrum acquired from the marked points shown in TEM image.

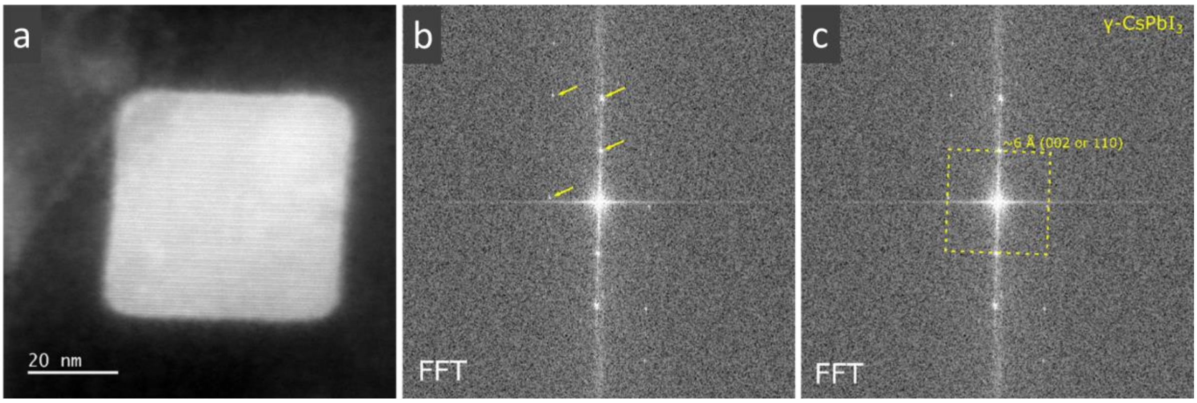

Supplementary Fig. 34. a Lattice-resolved ADF-STEM image of a FIB lamella showing a $CsPbI_3$ nanocrystal in a_gZIF-62. **b, c** The fast Fourier transform (FFT) of the crystal image was indexed to γ-$CsPbI_3$ based on the approximately square pattern of spots at ~6 Å (real-space spatial frequency). Annotations in yellow highlight the position of spots and their approximately square symmetry.

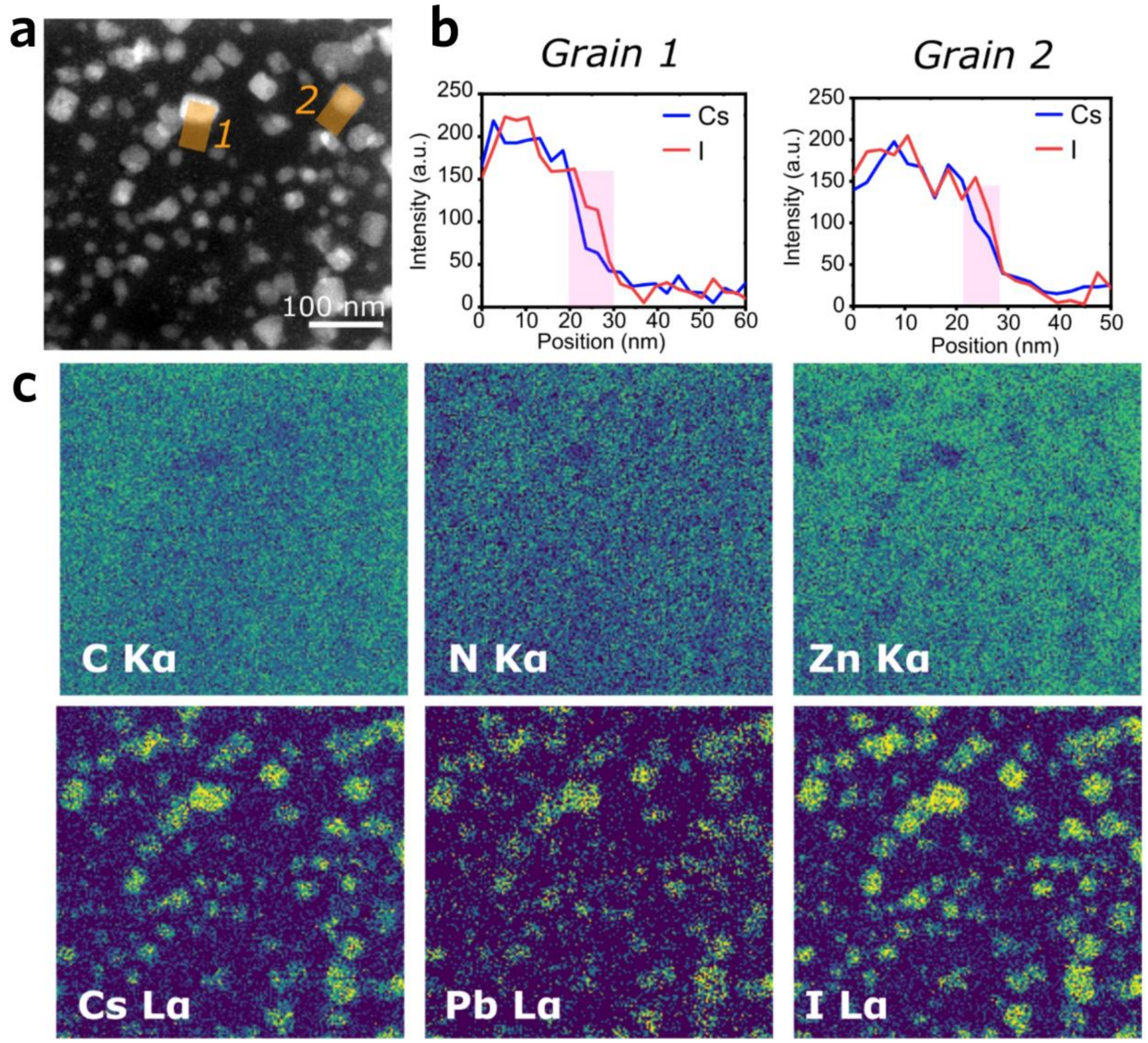

Supplementary Fig. 35. STEM-EDS analysis of $CsPbI_3$ nanocrystals in a_gZIF-62. a An overview ADF-STEM micrograph and **b** corresponding line profiles marked in orange. The line profiles show the Cs Lα and I Lα X-ray intensity at selected grains. The I Lα intensity extends further with the Cs Lα intensity decaying faster (magenta shaded regions), corroborating the ~5-10 nm further extension of I intensity observed in EELS beyond the detection of Cs in the nanocrystals. **c** EDS maps showing the major constituents of a_gZIF-62 (C, N, Zn) and $CsPbI_3$.

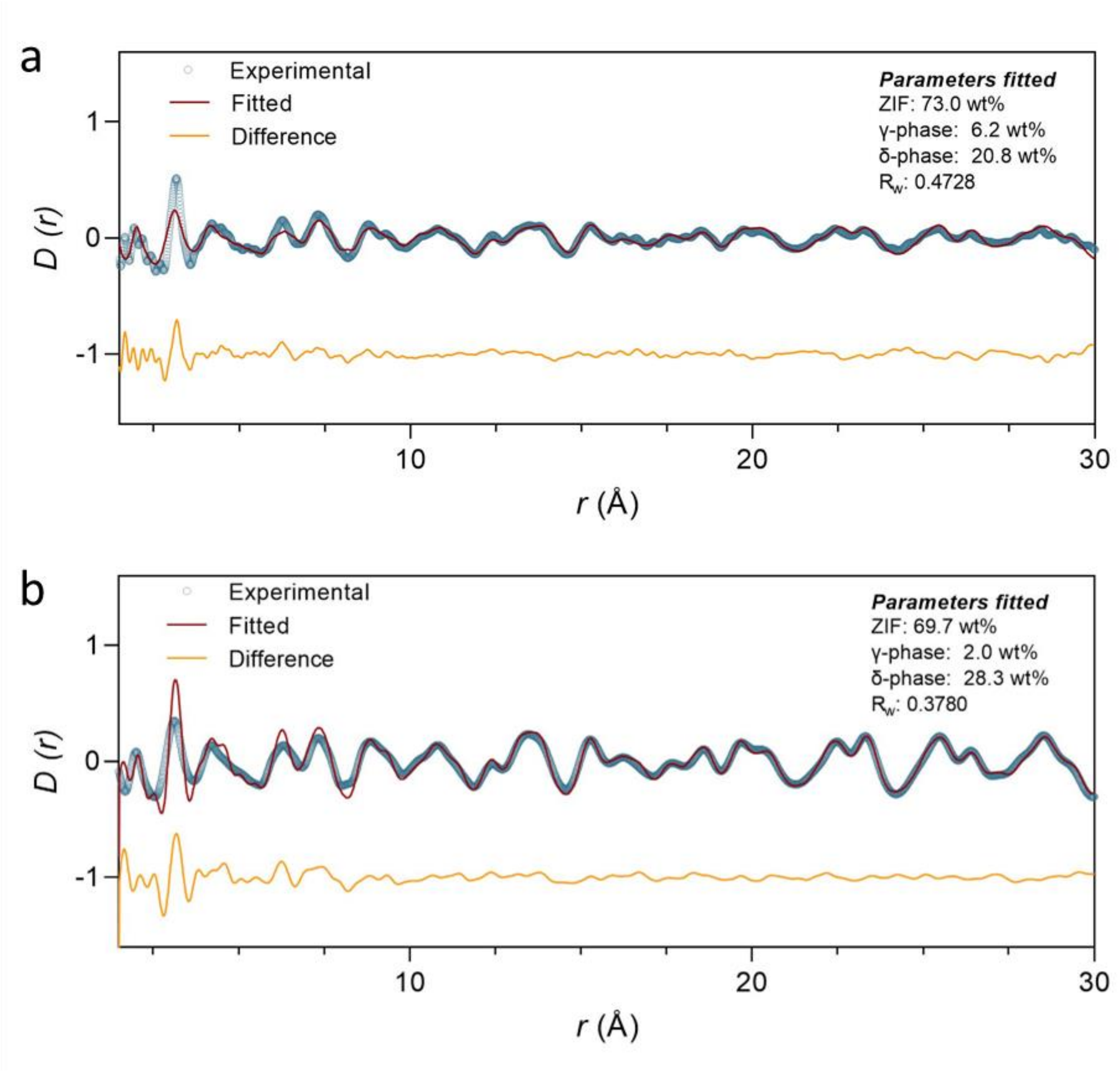

Supplementary Fig. 36. Atomic pair distribution functions for $(CsPbI_3)_{0.40}(a_gZIF\text{-}62)_{0.60}$ composites sintered at **a** 325 and **b** 350 °C. Pair distribution function *D(r)* calculated *via* Fourier transform of the X-ray total scattering function.

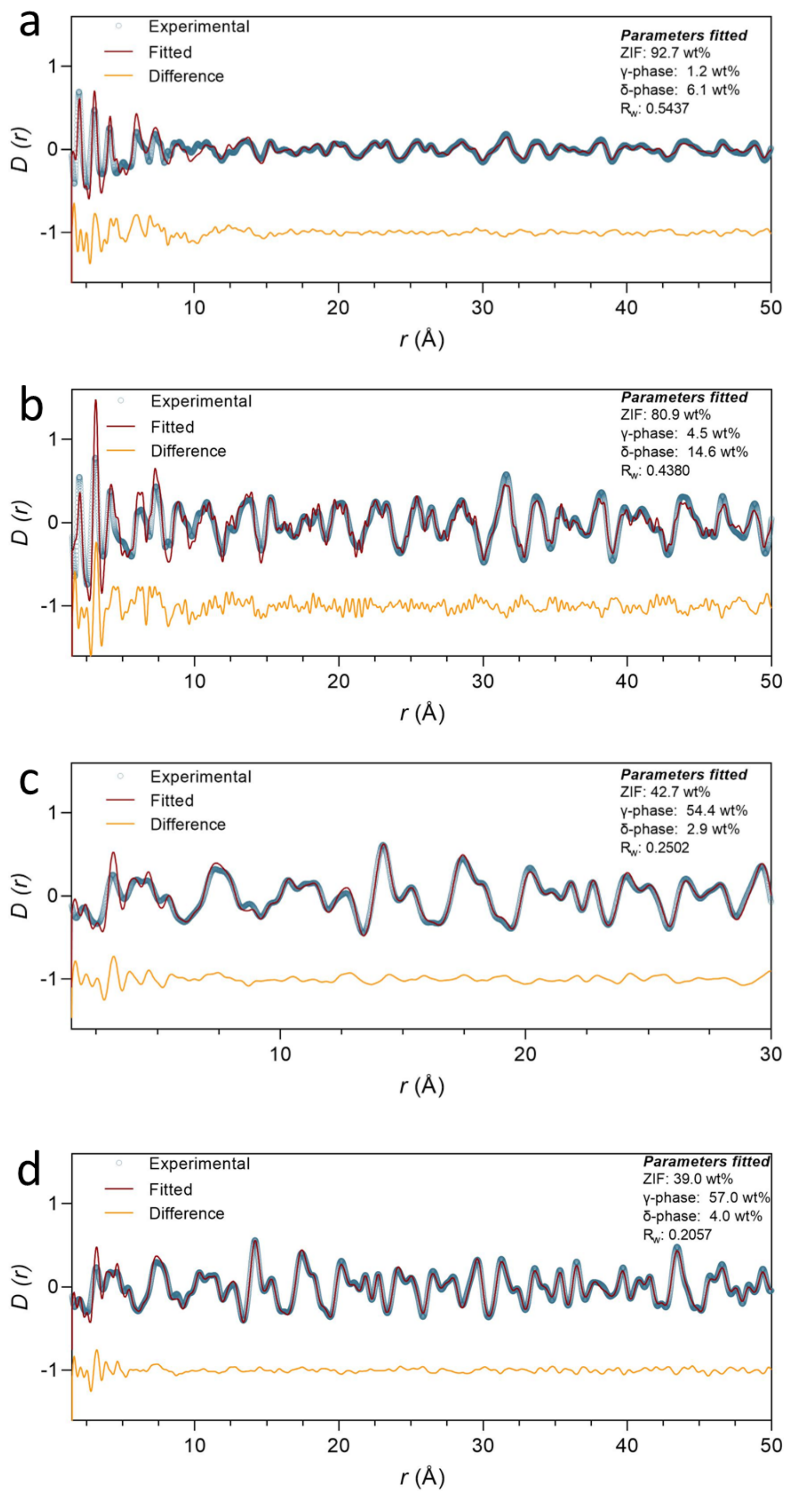

Supplementary Fig. 37. Atomic pair distribution functions for composite sintered at 350 °C with different $CsPbI_3$ loadings. **a** 10 wt%, **b** 25 wt%, **c** 70 wt% and **d** 85 wt%.

Supplementary References

1 Marronnier, A. *et al.* Anharmonicity and Disorder in the Black Phases of Cesium Lead Iodide Used for Stable Inorganic Perovskite Solar Cells. *ACS Nano* **12**, 3477-3486 (2018).

2 Hou, J. *et al.* Halogenated Metal-Organic Framework Glasses and Liquids. *J Am Chem Soc* **142**, 3880-3890 (2020).
